# Single‐Particle Insights Into the Electronic Structure and Enhanced Stability of CsPbBr_3_/FAPbBr_3_ Core/Crown Nanoplatelets at the Nanometer Scale

**DOI:** 10.1002/smll.202514675

**Published:** 2026-04-21

**Authors:** Shaocheng Shen, Anuraj S. Kshirsagar, John Michael Lane, Chenguang Zhang, Haixin Zhang, Kedar Hippalgaonkar, Neeraj Rai, Mahesh K. Gangishetty, Kun Wang

**Affiliations:** ^1^ Department of Chemistry University of Miami Coral Gables Florida USA; ^2^ Department of Chemistry Mississippi State University Mississippi State USA; ^3^ Dave C. Swalm School of Chemical Engineering and Center for Advanced Vehicular Systems Mississippi State University Mississippi State USA; ^4^ School of Materials Science and Engineering Nanyang Technological University Singapore Singapore; ^5^ Department of Physics University of Miami Coral Gables Florida USA; ^6^ Institute of Materials Research and Engineering Agency For Science Technology and Research (A*STAR) Singapore Singapore; ^7^ Department of Physics and Astronomy Mississippi State University Mississippi State USA

**Keywords:** core‐crown perovskite nanocrystals, electronic structure, scanning tunneling spectroscopy, stability enhancement, trap states

## Abstract

Colloidal perovskite nanoplatelets (NPLs), despite their exceptional optoelectronic properties, face significant challenges due to their intrinsic instability and trap‐assisted non‐radiative recombination. Although many studies employing surface engineering, such as selecting ligands or coating to passivate defects, have demonstrated improved optoelectronic properties, these enhancements are typically inferred from bulk‐ensemble measurements; the effects at the single‐particle level remain elusive. Here, we conduct single‐particle‐level studies using scanning tunneling spectroscopy (STS) on a unique core–crown system, CsPbBr_3_@FAPbBr_3_ NPLs, where the lateral surfaces of the CsPbBr_3_ core are coated with an FAPbBr_3_ crown. Experimental density‐of‐states (DOS) analysis reveals a 47% reduction in deep‐trap states in core‐crown NPLs compared to core‐only NPLs, consistent with nearly two‐fold enhancements in photoluminescence quantum yields. Progressive *I*–*V* sweep measurements demonstrate superior electrical stability in core‐crown NPLs, preserving band structure with minimal degradation, while core‐only NPLs exhibit rapid bandgap shrinkage and trap formation. Density functional theory (DFT) calculations indicate that FA incorporation distorts Pb octahedral lattice, widening the bandgap. This study elucidates how surface engineering modulates charge localization, passivates defects, and enhances stability at the single‐particle level. Moreover, by uncovering bias‐induced trap states in single unpassivated NPLs, this study establishes a precise, robust approach for characterizing emerging perovskite nanocrystals.

## Graphical abstract

Single‐particle‐level investigation using scanning tunneling spectroscopy reveals electronic structure and type‐I band alignment of individual CsPbBr_3_@FAPbBr_3_ core‐crown nanoplatelet. Crown passivation suppresses deep trap states by 47% and markedly improves electrical stability under repeated bias sweeps. Our results suggest lateral surface engineering as a powerful route to stabilize perovskite nanocrystals at the nanoscale.

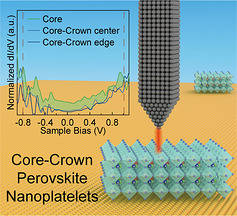

## 1 Introduction

Colloidal perovskite nanocrystals have garnered increasing interest in recent years owing to their pronounced quantum‐confinement effects, which enable bright luminescence with high photoluminescence quantum yields (PLQYs), emission spectra with narrow full‐width half maximum (FWHM), and tunable emission across the visible spectrum. These promising properties render perovskite nanocrystals ideal candidates for next‐generation optoelectronic technologies, including light‐emitting diodes (LEDs) [1, 2], lasers [3, 4], solar cells [5, 6], and photodetectors [7]. Despite these advantages, intrinsic instability and surface defects pose significant challenges, leading to non‐radiative recombination, reduced efficiency, and accelerated degradation, thereby limiting device efficiency and longevity in practical applications [8, 9]. To address these limitations, various surface passivation strategies have been explored using ligands, such as amines [10, 11], metal salts [12, 13], sulfonates [14, 15], and phosphonic acids [16, 17]. Recently, inspired by II‐VI family like CdSe/CdS quantum dots [18, 19], we developed type‐I core‐crown heterostructures comprising a CsPbBr_3_​ core laterally passivated by an FAPbBr_3_​ crown [20]. These type‐I core‐crown configurations facilitate carrier confinement in the core, enhancing efficient radiative recombination, while effectively passivating surface traps with crown layers [21]. The crown layer also provides structural protection of the perovskite core, which mitigates its susceptibility to degradation [22, 23]. Furthermore, the anisotropic passivation in core‐crown structures preserves 1D spatial quantum confinement, resulting in stable blue emission with enhanced optical properties compared with both core‐only structures [8, 20].

While the abovementioned strategies have advanced the development of stable and high‐performance materials, key insights into their intrinsic optoelectronic behavior, including local energy alignment [24, 25], charge carrier confinement [26], and distribution of surface trap states [27, 28, 29], have remained inaccessible through conventional ensemble measurements in bulk, such as PLQYs and device performance metrics [30, 31, 32, 33, 34]. As the effective material dimension scales down to a few nanometers, and due to the sample heterogeneity, the ensemble measurements may not necessarily reflect the local effects at the nanometer scale, thus hindering the understanding of underlying mechanisms [35].

To gain insights at the nanoscale, past studies have used scanning tunneling microscopy and spectroscopy (STM/STS) to probe local surface morphology, electronic inhomogeneity, and defect‐related states, particularly in halide perovskite thin‐film systems and extended crystalline surfaces [31, 32, 36, 37, 38, 39]. While these works have provided valuable insights into perovskite surface and interfacial physics of continuous films, globally modified nanocrystals, or large‐scale heterostructures, direct access to local effects in an individual laterally engineered perovskite nanocrystal remains experimentally limited. Spatially resolved single‐particle level investigation is thus essential for probing fundamental local phenomena in strongly quantum‐confined perovskite nanocrystals, enabling deeper mechanistic insights and paving the way toward targeted design optimization.

Lateral core‐crown nanoplatelet (NPL) heterostructures spatially expose the core and crown regions for direct experimental access, rendering them ideal for single‐particle level experimental investigation. In this work, we systematically investigate the electronic properties and stability of CsPbBr_3_/FAPbBr_3_ core‐crown NPLs. Specifically, combining in situ controlled material growth, STS, and DFT calculations, we seek to answer several key questions that have remained elusive: 1) What is the band structure of a single CsPbBr_3_/FAPbBr_3_ core‐crown NPL and how does it compare with theoretical predictions and ensemble measurements; 2) To what extent do the surface trap states reduce upon crown formation and passivation? Can these trap states be experimentally quantified in a single NPL? 3) Will the passivation, such as the presence of a crown layer, improve the stability of a single NPL, and if so, by how much? Our study provides direct experimental evidence of the type‐I band alignment in CsPbBr_3_/FAPbBr_3_ core‐crown NPL by identifying the positions of valence band maximum (VBM) and conduction band minimum (CBM) at a single‐particle level. Additionally, DOS analysis based on the STS reveals a reduction of the deep trap states by 47% within a single core‐crown NPL compared to core‐only structures, offering quantitative insights into the enhancement of ensemble PLQYs. To assess the stability of core‐crown NPLs, we monitor the variation of DOS upon continuous *I*–*V* sweeps on single core‐crown NPLs, revealing their enhanced stability to electrical degradation. Further, band structure and partial density of states (PDOS) indicate that the incorporation of FA^+^ induces Pb octahedral lattice distortion, resulting in a small widening of the bandgap. This study provides the first experimental evidence at a single‐particle level on how surface passivation governs charge localization and enhances stability in core‐crown NPLs, and how trap states emerge under applied bias in NPLs (unpassivated), offering fundamental insights for rationally designing more durable and defect‐tolerant perovskite nanocrystals.

## 2 Results and Discussion

**FIGURE 1:**
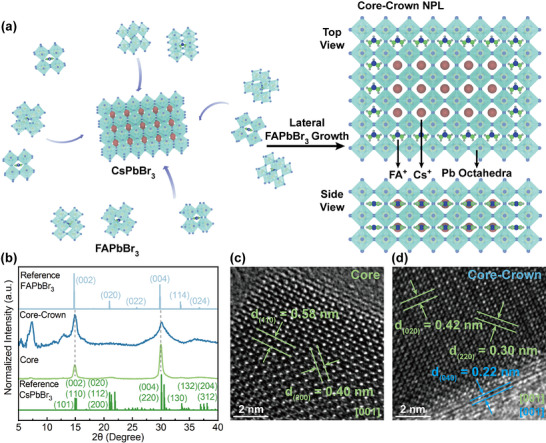
Synthesis and structural characterization of core and core‐crown NPLs. (a) Schematic of the lateral passivation dynamic process leads to CsPbBr_3_/FAPbBr_3_ core‐crown NPLs formation. (b) Powder XRD patterns of core and core‐crown NPLs. (c,d) HRTEM images of (c) core NPLs, (d) core‐crown NPLs.

CsPbBr_3_/FAPbBr_3_ core‐crown NPLs were synthesized using a room‐temperature ligand‐assisted reprecipitation (LARP) method with in situ growth control, as detailed in our previous work and in the Supporting Information [20]. The schematic of the formation of core‐crown NPLs is depicted in Figure 1a. Briefly, CsBr and PbBr_2_ precursor prepared in DMF was added to toluene containing a mixture of organic ligands (oleic acid and oleylamine), initiating the nucleation and growth of CsPbBr_3_ core NPLs. Subsequently, an FA^+^ precursor solution was introduced to promote the lateral growth of a thin FAPbBr_3_ crown layer. To prevent the overgrowth of NPLs, ethyl acetate was employed to quench the reaction. The final NPLs were purified using a multi‐step process, yielding high‐quality, monodisperse structures with uniform thickness and blue emission.

To confirm chemical composition and phase purity, the NPLs were characterized by X‐ray photoelectron spectroscopy (XPS) (Figures S1 and S2) and powder X‐ray diffraction (pXRD) (Figure 1b). Atomic concentrations for C, O, and N confirm the presence of ligands (Tables S1 and S2). pXRD patterns of CsPbBr_3_ NPLs and CsPbBr_3_/FAPbBr_3_ core‐crown NPLs correspond to an orthorhombic crystal phase. The coating of FAPbBr_3_ induces several new diffraction peaks, consistent with our previous report [20]. High‐resolution transmission electron microscopy (HR‐TEM) (Figure 1c,d; Figure S3) confirms the platelet morphology of the core and core‐crown NPLs, revealing surface facets of atomically thin ‘*flat’* NPLs with interplanar distance of 0.40 and 0.58 nm corresponding to the (200) and (110) crystal planes (intersecting with 45° angle) of orthorhombic CsPbBr_3_ (Figure 1c). The FAPbBr_3_ crown growth around the CsPbBr_3_ core exhibits a well‐defined interface (Figure 1d). The core regions of core‐crown NPLs show interplanar distances of 0.42 and 0.30 nm corresponding to (020) and (220) crystal planes of orthorhombic CsPbBr_3_. Whereas the crown regions of core‐crown NPLs exhibit the *d*‐spacing of 0.22 nm that corresponds to the (040) crystal plane of orthorhombic FAPbBr_3_.

**FIGURE 2:**
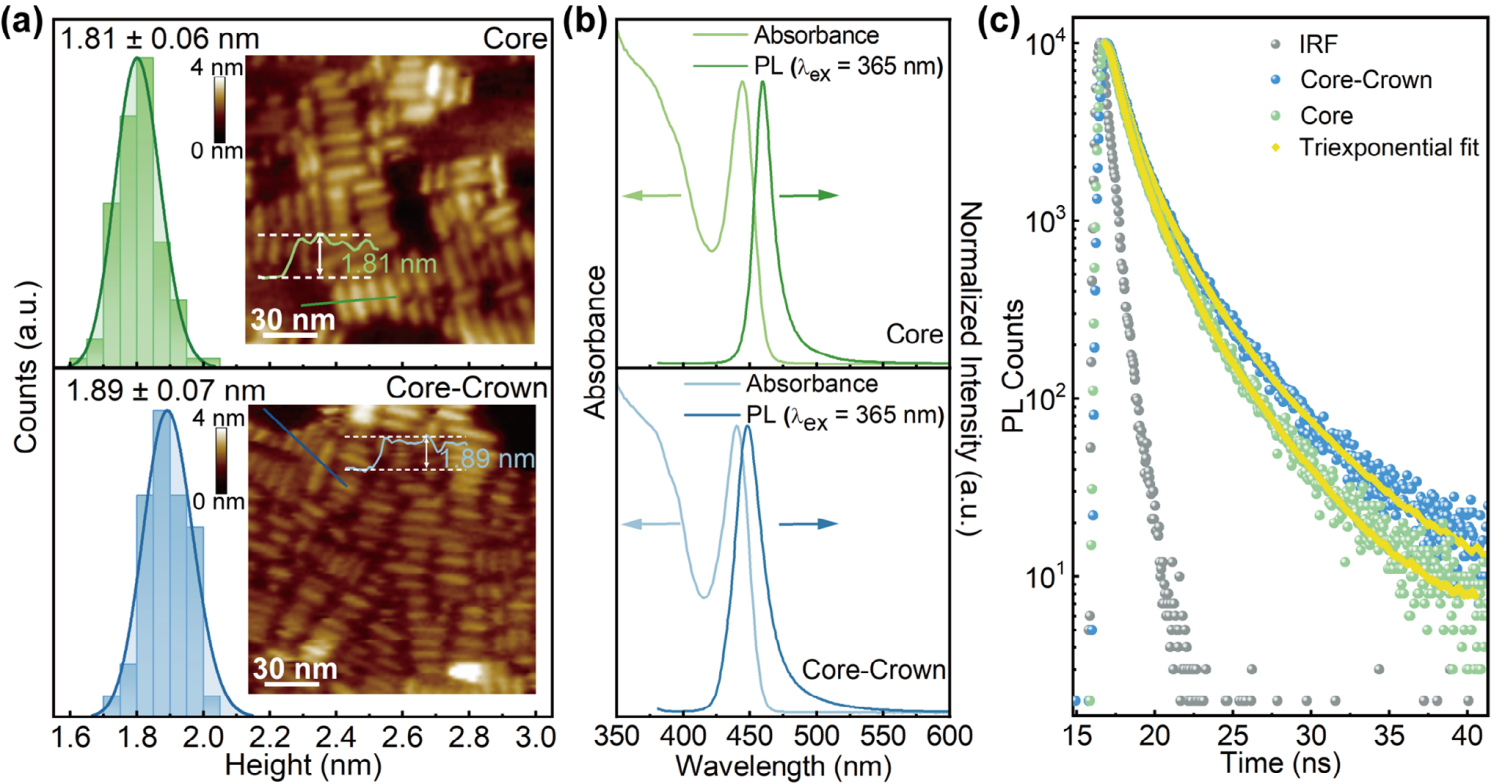
Evidence of 1D quantum confinement and optical properties of core and core‐crown NPLs. (a) AFM height histograms of core and core‐crown NPLs. The corresponding AFM height images, cross‐section, and height profiles are shown as insets. (b) UV–vis absorption and PL spectra for core and core‐crown NPLs. (c) Time‐resolved PL decay dynamics measured at room temperature for corresponding emission peak in panel (b).

To verify the 1D spatial confinement and to quantify the height and lateral dimensions of both core and core‐crown NPLs, atomic force microscopy (AFM) imaging was performed by depositing them on an Au(111) surface. As shown in Figure 2a and Figure S4, the NPLs are uniformly distributed on the surface, and statistical analysis based on over 100 individual NPL yields thickness values of 1.81 ± 0.06 nm for core NPLs and 1.89 ± 0.07 nm for core‐crown NPLs. The thickness is significantly smaller than the excitonic Bohr diameter (∼7 nm in bulk CsPbBr_3_) [40], confirming strong quantum confinement of the NPLs. The uniform thickness distribution across samples, regardless of composition variations, further supports the lateral growth of FAPbBr_3_ crown on the CsPbBr_3_ core. Considering the thickness of a single [PbBr_6_]^4−^ octahedral layer is approximately 0.6 nm [41, 42], these height profiles indicate the formation of three monolayer (ML) nanoplatelets. A slight difference in the thickness of core and core‐crown NPLs could be attributed to the stacking disorder and the surface effects [43].

As shown in Figure 2b, the anisotropic quantum confinement of the NPLs results in strongly blue‐shifted photoluminescence (PL) and UV–vis absorption spectra. The emission peak energy, Stokes shift, and full width at half maximum (FWHM) of core‐only and core‐crown NPLs are listed in Table S3. The core‐crown NPLs exhibit a slightly larger bandgap than their core‐only counterparts, which can be attributed to surface passivation and compositional modulation induced by FA^+^ incorporation. Consistently, XPS analysis reveals a lower Cs:Pb atomic ratio (Figure S5), reflecting the competing incorporation of FA^+^ and Cs^+^ ions for A‐site occupancy during growth.

**FIGURE 3:**
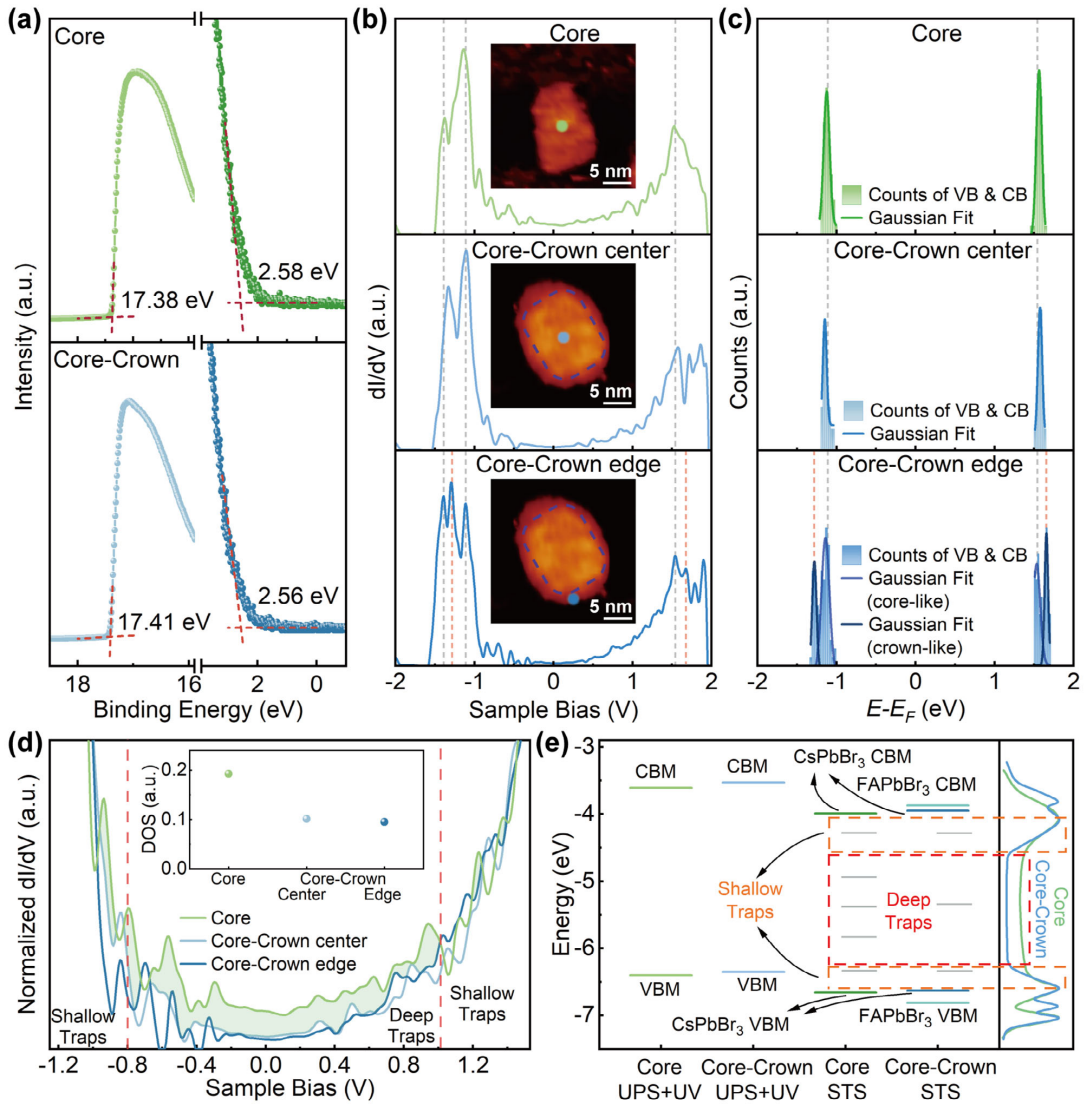
DOS characterization and energy level alignment for type‐I band structure. (a) UPS of core and core‐crown NPLs. (b) dI/dV spectra acquired from center of core NPLs and both center and edge of core‐crown NPLs. Gray dashed lines illustrate the VBM, CBM, and additional dominant peak at VB side. The orange dashed lines represent the VBM and CBM of FAPbBr_3_, respectively. Representative STM images of core and core‐crown NPLs are shown as insets. The purple dashed frames in core‐crown NPLs suggest the interface between core and crown. The dots on the NPLs demonstrate the STM tip position for dI/dV spectra acquisition. (c) Histograms of VB and CB for center of core NPLs and both center and edge of core‐crown NPLs. The gray and orange dashed lines represent the VBM and CBM of CsPbBr_3_ and FAPbBr_3_, respectively. (d) Normalized dI/dV spectra of the bandgap region shown in (b). The dashed lines separate the shallow trap states and deep traps region. The shallow green area shows the decreasing of DOS from core to core‐crown NPLs. The integration of dI/dV spectra for three scenarios in the deep trap states region is shown as inset. (e) Schematic representations of the band diagram via the synergic of UPS and UV–vis absorption and STS for core and core‐crown NPLs. The shallow trap states and deep trap states are marked by orange and red dashed frames, respectively.

In a core‐crown NPL, it is expected that the formation of type‐I core‐crown structure reinforces the radiative recombination by confining charge carriers within the core and hindering nonradiative channels by refilling the traps states [21]. This effect contributes to an increase in PL lifetime [8, 44]. As displayed in the PL and UV absorption spectra (Figure 2b), the core‐crown NPLs yield a smaller Stokes shift compared with that of the core‐only NPL. Time‐resolved PL (TRPL) spectra (Figure 2c; Table S4) display that despite similar decay profiles with the core‐only sample, the core‐crown NPLs exhibit an increasing decay lifetime, suggesting a reduction in defect states and well‐passivated surfaces. To further correlate these optical observations with the electronic structure, ultraviolet photoelectron spectroscopy (UPS) was performed to probe the valence band position and work function. As displayed in Figure 3a and Figure S6, the UPS measurements show a work function (Φ) of 3.82 eV for core‐only NPLs and 3.79 eV for core‐crown NPLs, with corresponding ionization energy (IE) of 6.40 and 6.35 eV, respectively. These results are consistent with a recent report [45] indicating that both NPL types exhibit similar electronic structures and comparable ligand‐induced surface dipoles.

However, these ensemble measurements face difficulty in accessing local electronic structure and energy alignment landscape between the core and crown at the nanoscale. Furthermore, how trap‐state reduction contributes to the electronic properties of individual NPLs and their optoelectronic performance is largely unexplored. In addition, experimental evidence of type‐I band alignment within individual core‐crown NPLs has remained unattainable to date. To quantitatively interrogate the type‐I band structure at the single‐particle level, we employed room temperature (19°C) STS, a spatially resolved approach, to distinguish the core and crown energy profiles and investigate their electronic structures in situ. The STS measurements were performed by hovering the STM tip (Pt/Ir) above single NPLs at designated locations (i.e., core or crown regions), with the initial set‐point established at 1 nA and 0.1 V prior to the feedback‐off current‐voltage (*I*–*V*) sweep. Then, the slope of the *I*–*V* curve at each voltage (*dI*/*dV*) vs. sample bias spectra of the single core and core‐crown NPLs were acquired, providing energy‐dependent DOS of the NPLs. In an STS spectrum, the relation between *dI*/*dV* and sample bias is described as:

$$\frac{dI}{dV}\;\propto \;\rho_{s}\left(E_{F}-eV\right)$$

where ρ_*s*_ is the DOS of the sample and *EF* is the Fermi energy [46]. This equation is under the assumption that the DOS of the tip is constant. More generally, the tunneling current can be written as,

$$I\left(V\right)\propto \;\int \rho_{s}\left(E\right)\rho_{t}\left(eV-E\right)T\left(E,V,z\right)dE$$

where ρ_*s*_ and ρ_*t*_ are the density of states of the sample and tip, respectively, and *T*(*E*, *V*, *z*) is the tunneling matrix element, which depends on energy, bias, and tip‐sample separation z [47]. Under identical tunneling conditions, the measured *dI*/*dV* spectra can therefore be used as a relative probe of the sample electronic structure rather than as an absolute DOS measurement, because the tip DOS enters as a common first‐order factor in the comparative analysis of the core‐only and core‐crown NPLs. A more detailed formal analysis of this comparative approximation, including the role of the tip DOS and tunneling transmission, is provided in Section S5.3. On the other hand, the *dI*/*dV* spectra also reveal the valence band (VB) and conduction band (CB) edges of NPLs as the first peaks on either side of the Fermi energy, [29, 38] which is aligned at 0 V. The positive sample bias probes the unoccupied states of the sample, and thus, the first peak indicates the CB edge of the core or core‐crown NPLs. Conversely, a negative sample bias probes the occupied states, with the first peak representing the VB edge of the core or core‐crown NPLs.

In contrast to the uniform distribution of electronic states in core NPL (inset top panel of Figure 3b), STM image of the core‐crown NPLs (inset middle and bottom panels of Figure 3b) reveal a clear boundary between two regions with the center region of the NPL showing more DOS (brighter) and the outer shell displaying less electronic states (darker), which can be attributed to the core region at the center and the crown region at the outer edge. To determine the DOS distribution, the STM tip was purposely positioned at the center of both core and core‐crown NPLs, as well as at the edge of the core‐crown NPLs. The DOS was then extracted from the corresponding *dI*/*dV* spectra in Figure 3b by averaging results from independent measurements on 60–80 NPLs (Figures S7 and S8). Specifically, for each measurement location category (e.g., core‐only center, core‐crown center, and core‐crown edge), one *I*–*V* sweep was acquired from a fresh, unmeasured NPL. The representative dI/dV spectra shown in Figure 3b was obtained by first averaging the independently collected *I*–*V* curves and then numerically differentiating the resulting average *I*–*V* curve. The STS spectra exhibit several distinct and well‐defined peaks near both VB and CB edges, consistent with the presence of discrete electronic states expected in quantum‐confined systems [48, 49, 50]. Although interfacial charge transfer, screening, or partial hybridization at the Au(111) interface may influence the absolute band‐edge alignment referenced to the Au(111) Fermi level, the spectra measured on the NPLs exhibit pronounced and reproducible band‐edge features with a finite bandgap. These features indicate that the NPLs remain sufficiently electronically decoupled from the substrate to preserve their semiconducting spectral characteristics.

As expected, similar DOS features were obtained at the center for both core and core‐crown NPLs due to the identical chemical composition of CsPbBr_3_. Two dominant peaks were identified at VB, with −1.13 and −1.11 eV as VBM with respect to the Fermi energy level for core and core‐crown NPLs, respectively. The CBM was observed at 1.53 and 1.59 eV for core and core‐crown NPLs, respectively. The slight broadening (∼0.04 eV) of the bandgap observed from STS aligns with the blueshift observed in UV absorption. Additionally, the increased relative intensity of the two dominant peaks at VB and additional peaks at CB indicate that electrons are more confined within the VB and CB states of the CsPbBr_3_ core within the core‐crown structure compared to CsPbBr_3_ core‐only NPLs. While distinct DOS features were detected at the edge of the core‐crown NPLs, attributed to the compositional variation from Cs^+^ to FA^+^. The additional peaks in STS spectra at the edge correspond to VBM and CBM of FAPbBr_3_, located at −1.27 and 1.66 eV, respectively, relative to the Fermi energy level. To evaluate the robustness of extracted band‐edge values, additional statistical analysis was performed by first extracting the VBM and CBM values from individual dI/dV spectra and then compiling them into histograms (Figure 3c; Figures S9 and S10). The extracted band‐edge values are summarized in Tables S5–S8. The histogram analysis reproduces the same overall trends obtained from the averaged *I*–*V* derived spectra (Figure S11), namely a bandgap widening at the center of the core‐crown NPLs and two distinct sets of band edges at the core‐crown edge. These results confirm that the observed band‐edge positions and their differences are statistically robust and originate from the distinct variation in chemical composition across different regions.

Notably, the reduction of local defects and surface traps can be directly illustrated from STS spectra, offering a unique opportunity to quantitatively reveal the energy landscape of the trap states in a single NPL. Figure 3d represents the DOS within the bandgap by stacking the STS spectra in Figure 3b together. The shaded green region highlights the decrease of DOS from core NPLs to core‐crown NPLs, both at the center and edge. The trap states can be classified into shallow trap states and deep trap states. Shallow trap states only require a relatively low thermal energy (a few *k_B_T*) to escape back into the excited transport states (corresponding to VBM and CBM) [51], which are generally considered harmless. In contrast, deep trap states require higher activation energy, thus leading to the primary efficiency loss [51]. Since the trap‐assisted recombination is primarily induced by states lying 0.3–0.6 eV above or below the transport states [52, 53, 54, 55], acting as deep trap states, our analysis focuses on the trap states within the range between two dashed lines (−0.8–1.0 eV) in Figure 3d. Here, this interval is used as a conservative operational window for bromide‐based perovskites to exclude near‐edge shallow/tail states and to quantify the more deeply located in‐gap spectral intensity in a consistent manner across samples [56, 57, 58]. It is evident that a significant reduction of the deep trap states was achieved upon the formation of the crown layer. For quantitative comparison of this deeper in‐gap spectral contribution, each dI/dV spectrum was normalized by the intensity of its principal VB peak before integration over the selected energy window. Specifically, the DOS within the deep trap states region decreases by more than 47%, while no significant variations are observed in the shallow trap state region. The observed trap states suppression agrees well with the nearly two‐fold improvement in thin film PLQYs in our previous report (same batch of sample) due to the removal of surface dangling bonds through effective surface passivation [20]. These findings from single‐particle investigation, for the first time, establish the direct correlation between trap‐state reduction and PLQY enhancement, offering quantitative insight into how surface passivation impacts optoelectronic properties. Under our experimental conditions, this interpretation is not expected to be dominated by tip‐induced band bending (TIBB) or screening effects. As discussed in the Section S5.4, SEMITIP electrostatic simulations [59] together with UV‐assisted STS measurements indicate that any TIBB present is limited and does not alter the main band‐edge or trap‐state assignments (Figures S12–S18 and Tables S9 and S10).

By integrating UV absorption and UPS with STS results, we constructed the band diagram of core and core‐crown NPLs, as depicted in Figure 3e. The STS‐derived band‐edge positions were aligned using the Au(111) Fermi‐level value as the reference energy scale, while in UPS, the VBM is extracted from the ensemble VB onset on ITO within the vacuum‐level/work‐function framework. The discrepancies in energy levels from STS compared with the UPS and UV absorption are attributed to the interplay of deeper work function of Au(111) and the local surface effect, including interactions with the Au(111) substrate and TIBB [60, 61]. Moreover, by leveraging the intrinsic lateral geometry of the core‐crown heterostructure, our STS measurements enable direct interrogation of the type‐I core‐crown band structure within a single NPL, overcoming the limitations of prior experimental efforts that relied on inferring such band alignment from the electronic properties of its individual components.

**FIGURE 4:**
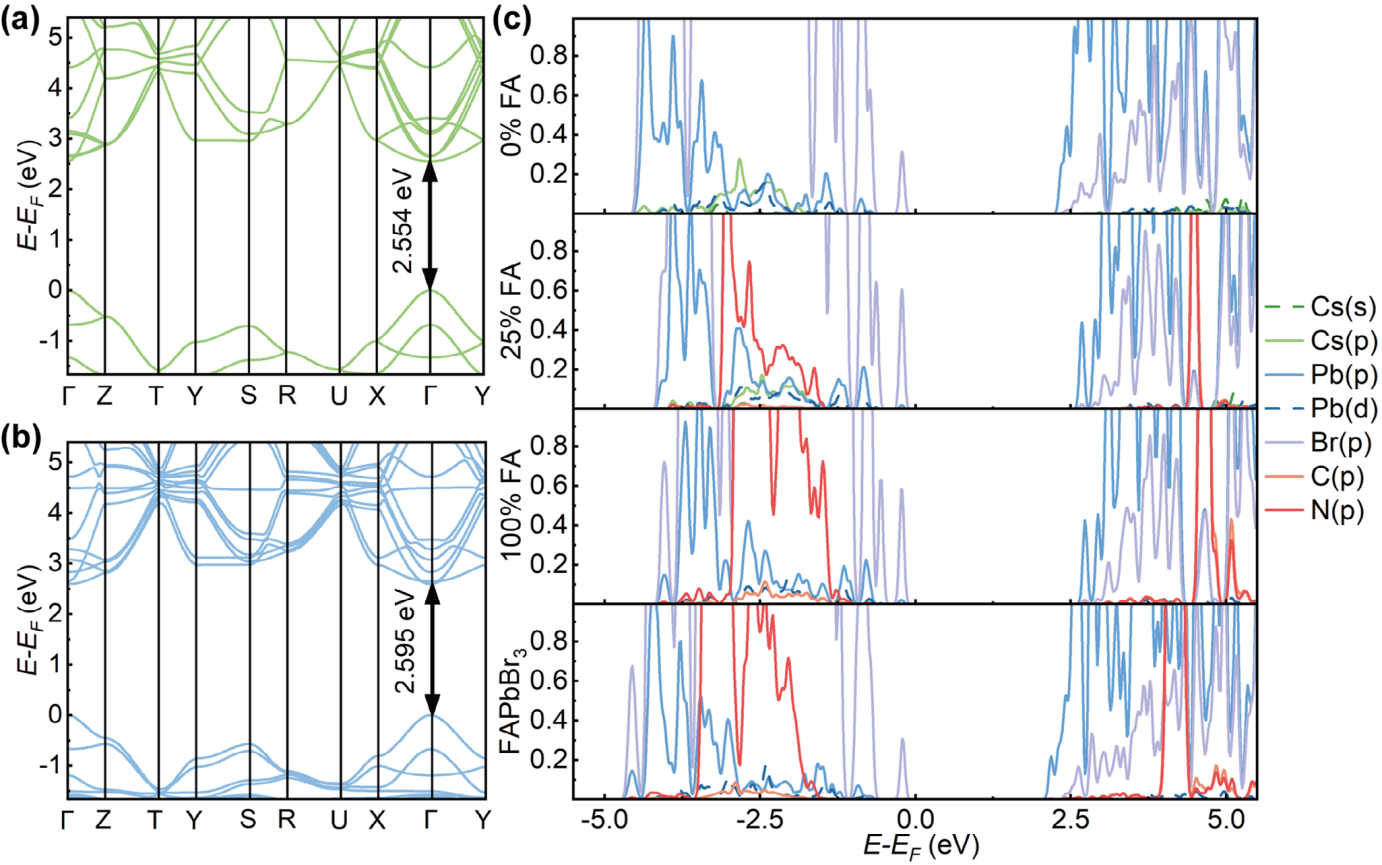
Band structure and the orbital contributions to the edge states as a function of FA concentration. (a) The band structure of orthorhombic CsPbBr_3_ (0% FA system) with a bandgap of 2.554 eV. (b) The band structure of the 25% FA system with a bandgap of 2.595 eV. (c) Partial density of states as a function of FA concentration, demonstrating the effect on the VBM and CBM, with orthorhombic FAPbBr_3_ included for comparison.

To gain a deeper insight into the role of FA incorporation, we carried out DFT calculations pristine and FA substituted orthorhombic CsPbBr_3_ lattice. Briefly, one or all Cs atoms were replaced by FA molecules in the unit cell, yielding a 25% FA substitution system and a 100% FA substituted system, respectively. Meanwhile, an orthorhombic FAPbBr_3_ lattice was also included for comparison (Figure S19). Given FA molecules are anisotropic in shape, the effect of molecular orientation was also investigated in the 100% FA system. Two models were considered: FA aligned along the a lattice vector (a‐aligned) and along the b lattice vector (b‐aligned) (Figure S19). The lattice constant elongates in the direction of FA alignment, due to the molecule's larger size compared with Cs^+^ (Table S11). While the PDOS remains nearly identical between the two orientations, the calculated band gaps differ by around 0.08 eV, highlighting the sensitivity of the Pb octahedral lattice to local distortions (Figures S20 and S21 and Table S12). In the orthorhombic FAPbBr_3_ phase we investigated, FA molecules preferentially align along the a axis. However, FA molecules are largely disordered in real NPLs, and the orientation effect is therefore expected to average out. In the 25% substitution model, the FA molecule is aligned along the b vector, therefore for clarity and consistency, we present the band structure and PDOS of the b‐aligned system, as shown in Figure 4. We have selected this alloy‐like model to maintain computational feasibility while having Cs^+^ to FA^+^ cation ratios comparable to the true experimental system. Although this alloy‐like model does not precisely mimic the core‐crown configuration used in the experiments, it offers critically relevant insights, such as how FA affects the Pb─Br electronic framework.

The DFT calculations show a bandgap increase (∼0.04∼0.13 eV) upon FA substitution (Figure 4a,b; Table S12), consistent with the experimental observation of slightly larger band gaps (i.e., blueshift) in core‐crown NPLs compared to core‐only systems. The PDOS remains largely unchanged with increasing FA content, (Figure 4c; Figure S22) confirming that the band edges are dominated by Pb(p/d) and Br(p) orbitals, while A‐site contributions are minor. Moreover, FA incorporation reduces the electron effective mass along the in‐plane Γ‐X and Γ‐Y directions while increasing it along Γ‐Z, reflecting enhanced delocalization within the NPL plane and stronger confinement along the thickness direction (Table S12). Taken together with the experimental observations, the theoretical results indicate that FA incorporation does not directly alter the band‐edge states, but rather distorts the Pb octahedral lattice while maintaining the 1D spatial quantum confinement, resulting in bandgap broadening in core‐crown NPLs.

**FIGURE 5:**
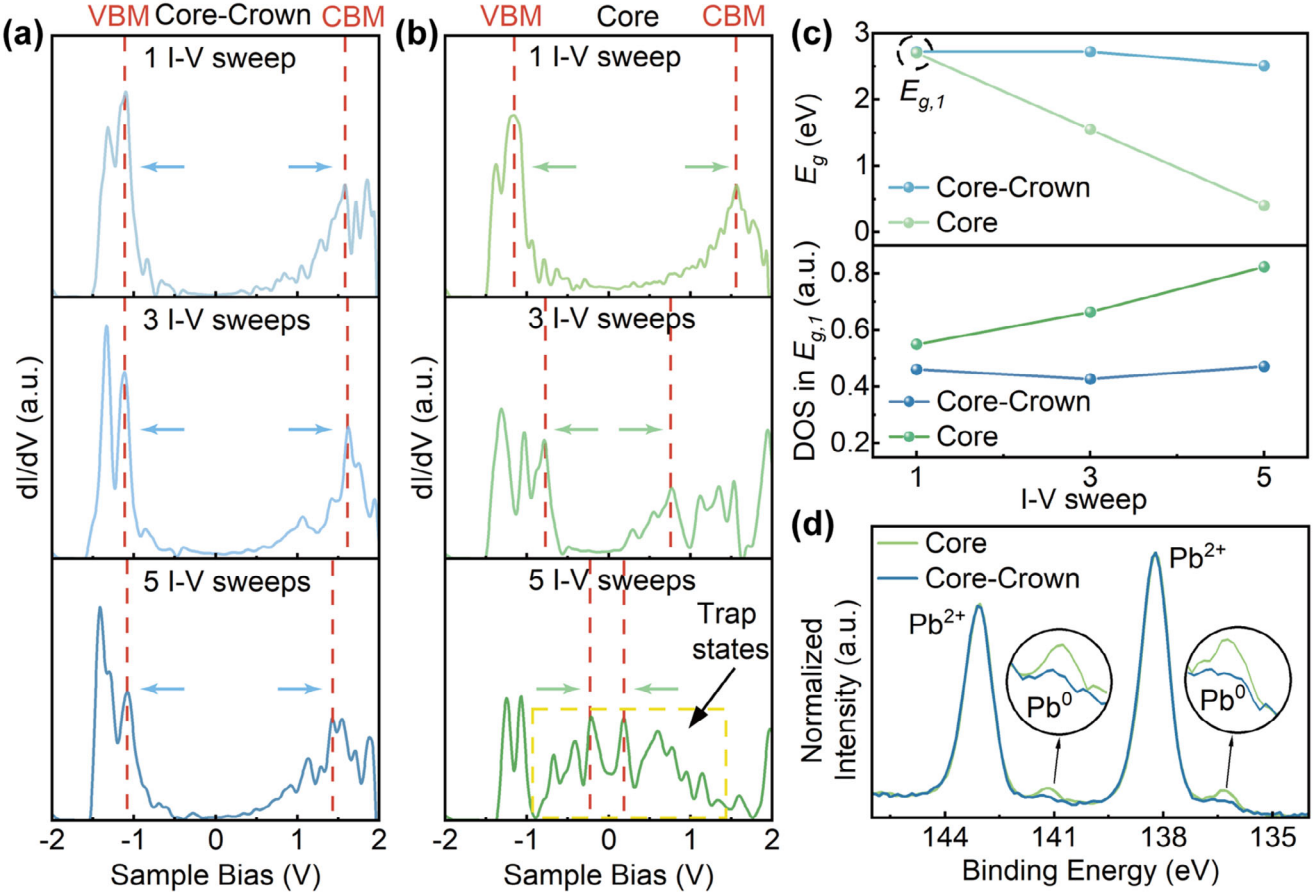
Core and core‐crown stability dynamics. (a,b) dI/dV spectra acquired from center of (a) core‐crown NPLs and (b) core NPLs under 1, 3, and 5 *I*–*V* sweeps, respectively. The red dashed lines represent the evolution of VBM and CBM for both core‐crown and core NPLs, respectively. The increased trap states after 5 *I*–*V* sweeps of core‐only sample are highlighted in yellow dashed frame. (c) The bandgap evolution and DOS which are integrated from the range of the bandgap determined from the first *I*–*V* sweep (*E_g,1_*) for both core and core‐crown NPLs under 1, 3, and 5 *I*–*V* sweeps, respectively. (d) High‐resolution XPS of Pb^2+^ and metallic Pb.

In type‐I core‐crown system, the NPLs are anticipated to exhibit enhanced stability compared to the core‐only NPLs [21]. To systematically evaluate the robustness of such an effect, we performed a series of STS measurements by continuously applying *I*–*V* sweeps to the same individual core or core‐crown NPLs, while monitoring the evolution of band structure and trap states. Continuous *I*–*V* sweeps emulate local electron injection into the NPLs, thus allowing us to interrogate the stability of core and core‐crown NPLs against electrical degradation. For consistency, we compared the *dI*/*dV* spectra of both core and core‐crown NPLs with same number of *I*–*V* sweeps. Figure 5a,b illustrate the STS spectra evolution when the core‐crown and core NPLs undergo 1, 3, and 5 *I*–*V* sweeps, respectively.

Upon exposure to 5 *I*–*V* sweeps, only a trivial bandgap shrinkage and lower DOS intensity and variations within the bandgap were observed in core‐crown NPLs, as shown in Figure 5a, confirming that the crown layer effectively shields the core from electronic degradation. The fact that DOS distribution remained intact indicates that core‐crown structures maintain their electronic structure despite prolonged electrons injection, further demonstrating their enhanced stability. In contrast, a significant bandgap shrinkage and a pronounced increase in DOS (or trap states, highlight in a dashed square at the bottom panel of Figure 5b) within the bandgap were observed for core NPLs, implying rapid degradation under electron injection (Figure 5c) that induced a transition from a semiconducting to metallic behavior. The pronounced DOS rise within the original bandgap suggests that core‐only structures are highly susceptible to electron‐induced defect formation, leading to progressive lattice destabilization (Figure 5c).

The electrical stability of core‐crown NPLs arises from several inter‐related factors. First, the passivation of FAPbBr_3_ crown layer prevents charge accumulation at defect sites, which are a primary trigger for degradation [62]. STS measurements confirm a reduction in deep trap states, indicating fewer defect sites for charge accumulation and thus a slower degradation rate. The Delta‐SCF calculations show that FA molecules preferentially accommodate excited charges, thereby alleviating direct perturbations to the Pb octahedral lattice and suppressing charge accumulation (Figure S23) [63]. Second, charge injection is known to induce halide migration in perovskites, leading to phase segregation and defect formation [64, 65]. Compared with core‐only structures where the migration pathways are more accessible, core‐crown interface acts as a halide migration energy barrier and simultaneously screens local electric fields that drive ionic motion [66, 67]. Although FA incorporation introduces slight local distortions, the combined passivation and charge‐redistribution effects dominate, resulting in suppressed trap formation and mitigated halide migration, thereby preventing charge‐induced phase segregation and defect formation. Third, without the protection of crown passivation, excess electrons destabilize the ionic [PbBr_6_]^4−^ octahedra lattice, which leads to structural degradation and the formation of byproducts, such as lead halides or metallic lead, causing transition from semiconducting to metallic properties [68]. Independent XPS measurements further show that the metallic Pb component (∼141 and ∼137 eV) is lower in the core‐crown NPLs than in the core‐only NPLs (Figure 5d). The atomic concentration ratio of Pb^0^ relative to all Pb species has a 25% reduction in core‐crown NPLs compared with core‐only NPLs (Figure S24). This result suggests that the core‐only NPLs have a higher intrinsic tendency toward Pb‐related reduction/degradation, which is consistent with their poorer electrical stability observed in the repeated‐sweep STS measurements. Lastly, excess charge can drive surface lattice distortion and ionic reorganization, generating defect sites that act as non‐radiative recombination centers and exacerbate the instability of core‐only NPLs (Figure 5c) [27, 69]. In contrast, in core‐crown NPLs, surface passivation suppresses such charge‐induced lattice distortions, reducing non‐radiative losses and preserving long‐term electrical stability [70]. Other factors involved in the STS measurements, including local electric field‐ or heating‐induced effects may also contribute to the degradation process, as reported in previous studies [71, 72, 73]. The different spectral evolution observed experimentally indicates that the core‐crown architecture more effectively suppresses these degradation pathways. These results highlight the potential of type‐I core‐crown engineering in addressing trap‐induced losses and material instability, paving the way for the development of more stable, high‐performance perovskite LEDs and photodetectors.

## 3 Conclusion

This study provides the first single‐particle‐level investigation of CsPbBr_3_/FAPbBr_3_ core‐crown NPLs, offering direct experimental evidence of type‐I band alignment, surface trap reduction, and stability enhancement. STS measurements confirm that both core and crown maintain distinct electronic energy levels, aligning with the type‐I band structure. Trap‐state reduction is quantified by a 47% decrease in DOS within the bandgap, consistent with the nearly two‐fold PLQY enhancement reported in ensemble measurements. Beyond trap suppression, progressive *I*–*V* sweep STS experiments reveal the superior stability of core‐crown NPLs under electron injection. While core‐only NPLs undergo bandgap shrinkage and significant in‐gap DOS increase, core‐crown NPLs retain their band structure with minimal changes. DFT calculations suggest that FA incorporation does not directly alter the orbital contributions to the band‐edge states, but instead induces distortions in the Pb─Br framework, thereby enhancing quantum confinement and accounting for the observed bandgap widening in core‐crown NPLs. By integrating STS, UPS, and optical characterization, this work bridges nanoscale electronic properties with macroscopic optoelectronic performance. These findings establish core‐crown engineering as an effective strategy for stabilizing perovskite NPLs, paving the way for next‐generation optoelectronic devices with improved efficiency and longevity.

## Author Contributions

K.W. and M.G. conceived the project. A.K. synthesized NPLs and performed optical spectroscopy measurements. S.S. performed STS measurements and contacted the UPS measurements under the supervision of K.W., M.G., and C.Z. characterized their structures via HR‐TEM under the supervision of K.H. and H.Z. conducted AFM measurements under the supervision of K.W. and J.M.L. conducted the theoretical calculations for the band structure and DOS information under the supervision of N.R., S.S., A.K., M.G., and K.W. discussed and analyzed the experimental data. S.S., A.K., J.M.L., N.R., M.G., and K.W. wrote the paper with input from all authors.

## Ethics Statement

The authors have nothing to report.

## Conflicts of Interest

The authors declare no conflicts of interest.

## Supporting information




**Supporting File**: smll73503‐sup‐0001‐SuppMat.pdf.

## Data Availability

The data that support the findings of this study are available from the corresponding author upon reasonable request.
