# Supplementary material for: Single‐Particle Insights Into the Electronic Structure and Enhanced Stability of CsPbBr3/FAPbBr3 Core/Crown Nanoplatelets at the Nanometer Scale
Source: Small. 2026 Apr 21;22(44):e14675. doi: 10.1002/smll.202514675 (PMC13450134; doi:10.1002/smll.202514675)
Supplement: Supplementary file 1 [file SMLL-22-e14675-s001.pdf]

Supporting Information

Single-Particle Insights into the Electronic Structure and Enhanced Stability of $CsPbBr_3/FAPbBr_3$ Core/Crown Nanoplatelets at the Nanometer Scale

*Shaocheng Shen[†1], Anuraj S. Kshirsagar[†2], John Michael Lane[†3], Chenguang Zhang[4], Haixin Zhang[5], Kedar Hippalgaonkar[4,6], Neeraj Rai[*3], Mahesh K. Gangishetty[*2,7], Kun Wang[*1,5]*

[1]Department of Chemistry, University of Miami, Coral Gables, FL 33146, United States.

[2]Department of Chemistry, Mississippi State University, Mississippi State, MS 39762, United States.

[3]Dave C. Swalm School of Chemical Engineering and Center for Advanced Vehicular Systems, Mississippi State University, Mississippi State, MS 39762, United States.

[4]School of Materials Science and Engineering, Nanyang Technological University, Singapore 639798, Singapore.

[5]Department of Physics, University of Miami, Coral Gables, FL 33146, United States.

[6]Institute of Materials Research and Engineering, Agency for Science Technology and Research (A*STAR), Singapore 138634, Singapore.

[7]Department of Physics and Astronomy, Mississippi State University, Mississippi State, MS 39762, United States.

† These authors contributed equally to this work.

* Correspondence should be addressed to Neeraj Rai (neerajrai@che.msstate.edu), Mahesh K. Gangishetty (mgangishetty@chemistry.msstate.edu) and Kun Wang (kunwang@miami.edu).

Table of contents

1. Materials and Synthesis 3
1.1 Materials and Chemicals 3
1.2 Synthesis of Core and Core-Crown Nanoplatelets (NPLs) 3
2. Optical Characterization 4
2.1 UV-Vis Absorption Spectroscopy 4
2.2 Photoluminescence (PL) Measurements 4
2.3 Time-Resolved Photoluminescence (TRPL) 4
3. Structural and Morphological Characterization 4
3.1 X-ray Diffraction (XRD) 4
3.2 High-Resolution Transmission Electron Microscopy (HR-TEM) 4
3.3 Atomic Force Microscopy (AFM) 5
4. Surface and Chemical States Characterization 5
4.1 X-ray Photoelectron Spectroscopy (XPS) 5
5. Electronic Characterization 5
5.1 Ultraviolet Photoelectron Spectroscopy (UPS) 5
5.2 Scanning Tunneling Microscopy and Scanning Tunneling Spectroscopy (STM/STS) 6
5.3 Assumptions in the Comparative Quantification of Trap-Related In-Gap Spectral Intensity 6
5.4 Tip-induced Band Bending (TIBB) and Screening Effects 8
6. Theoretical Calculations 13
7. Supplementary Tables 14
8. Supplementary Figures 20
9. References 39

1. Materials and Synthesis

1.1 Materials and Chemicals

Lead bromide (⩾ 98%), Cesium bromide (99.99%), oleic acid (90%), oleylamine (⩾ 98%), anhydrous N, N’-dimethyl formamide (DMF, 99.8%), ethyl acetate, octane (⩾ 99%), hexane (95%), anhydrous toluene (99.8%), and phenethylammonium bromide (PEABr, ⩾ 98%) were procured from SigmaAldrich. Formamidium bromide (FABr, 98%) was purchased from Ossila. All the chemicals were used as received without further purification.

1.2 Synthesis of Core and Core-Crown Nanoplatelets (NPLs)

The blue-emitting NPLs were synthesized using the ligand-assisted re-precipitation (LARP) method at room temperature. This involves the preparation of three different solutions at room temperature (25 °C). To prepare precursor solution “A” a mixture of CsBr (0.5 mM), and $PbBr_2$ (1.0 mM) dissolved in 20 mL of anhydrous DMF whereas precursor solution “B” was prepared by dissolving FABr (0.5 mM) and $PbBr_2$ (1.0 mM) in 20mL of DMF. In another vial, a solution containing oleic acid (100 μL) and oleyl amine (100 μL) in anhydrous toluene (10 mL) was prepared and labeled as “C”. To completely dissolve precursor salts in DMF, the solutions “A” and “B” were vigorously stirred for 30 min at room temperature. The solution “C” vortexed for 10 s at room temperature for homogeneous mixing of organic ligands. Subsequently for the synthesis of core-crown NPLs, first, 500 μL of solution “A” was added swiftly into solution “C”. Approximately after couple of seconds, the precursor solution “B” was quickly added in above mixture and then reaction was quenched by swift addition of ethyl acetate (12 mL). Next, the mixture was centrifuged at 5000 rpm for 5 min to remove impurities and larger platelets. The supernatant was collected separately in a 50 mL centrifugation tube and to this supernatant, oleic acid, oleyl amine, and PEABr (10 μL each) were added, and the mixture was vortexed for 5 s. Then NPLs reprecipitated by the addition of 15 mL of ethyl acetate and mixture was centrifuged at 7800 rpm for 15 min. The resulting precipitate was dispersed in 1 mL of anhydrous hexane and oleic acid and oleyl amine (2 μL each) were added for passivation. The NPLs were again reprecipitated using 2 mL ethyl acetate to remove excess ligands. The mixture was then centrifuged at 7800 rpm for 15 min, the supernatant was discarded, and the precipitate was re-dispersed in

anhydrous octane (0.5 mL). The same procedure was followed for the synthesis of $CsPbBr_3$ NPLs but only precursor solution "A" (1 mL) was used without addition of solution "B".

2. Optical Characterization

2.1 UV-Vis Absorption Spectroscopy

Varion Cary 100 Bio UV-visible spectrophotometer was used for UV-visible absorption measurements of colloidal solutions.

2.2 Photoluminescence (PL) Measurements

Photoluminescence spectra of colloidal solutions were measured using a 365 nm LED lamp coupled with an adjustable collimation adapter (ThorLabs) and QR Pro high-performance spectrometer coupled with optical fiber (Ocean Insight).

2.3 Time-Resolved Photoluminescence (TRPL)

TRPL measurements were carried out using a Deltaflex modular fluorescence lifetime system (Horiba Scientific) equipped with a 356 nm LED source. The best-fit parameters for PL decay dynamics of NPLs are shown in **Table S4**. The PL decay plots were fitted to a triexponential decay function, and the average lifetime (τ_{avg}) was calculated using the equation, $\tau_{avg} = (A_1\tau_1^2 + A_2\tau_2^2 + A_3\tau_3^2)/(A_1\tau_1 + A_2\tau_2 + A_3\tau_3)$. τ_1, τ_2, and τ_3 are lifetimes of tri-exponential decays with contributions A_1, A_2, and A_3, respectively.

3. Structural and Morphological Characterization

3.1 X-ray Diffraction (XRD)

Drop casted films on silicon substrates were used for the x-ray diffraction measurements using PROTO, AXRD benchtop powder diffraction system equipped with Cu Kα (1.54 Å) radiation.

3.2 High-Resolution Transmission Electron Microscopy (HR-TEM)

All samples for TEM study were prepared by drop casting the diluted solution with octane (ratio 1:3) on ultrathin lacey carbon support film (<3 nm) of 300 mesh Au grid (01824G, Ted Pella, Inc., CA, USA). TEM characterizations were performed on JEOL JEM ARM 200F equipped with a 4096×4096 pixels^2 OneView CMOS camera (Gatan, Inc., Pleasanton, CA, USA). TEM images

from many different areas and magnifications ranging from 200 kx to 350 kx were acquired using full camera resolution 4096×4096 pixels^2 with 1 sec exposure time. For this study, TEM was operated at 200 kV with emission current of 8 μA. Images were processed using standard microscopy software Gatan Digital Micrograph (DM).

3.3 Atomic Force Microscopy (AFM)

All samples for AFM studies were prepared by drop casting the diluted solution with octane (ratio 1:1) on freshly annealed gold (111) surface on mica. The AFM images were collected with Bruker Multimode 8 AFM under Tapping mode under ambient conditions. The probes used for the AFM imaging were Bruker NCHV-A probes with a spring constant of 42 N/m.

4. Surface and Chemical States Characterization

4.1 X-ray Photoelectron Spectroscopy (XPS)

The sample was prepared via spin-coating on ITO glass. XPS data was collected on a Kratos Supra+ system with a monochromatic Ka x-ray source operated at 150 W. A charge neutralizer was used to prevent charging when necessary and all spectra were corrected to the C 1s peak at 284.6 eV. Survey and high-resolution scans were acquired at pass energies of 80 eV and 20 eV, respectively, and the analyzed spot size was 300 x 700 um. Kratos ESCApe software was used for data analysis, Shirley baselines were used, and the relative sensitivity factors used for quantification were those from the ESCApe software. The binding energy (BE), full width half maximum (FWHM), relative sensitive factor (RSF) and atomic concentration of the corresponding element peaks for both core and core-crown NPLs samples are listed in **Table S1** and **S2**, respectively. The Pb 4f A and B are referring to the metallic Pb and oxidized Pb, respectively.

5. Electronic Characterization

5.1 Ultraviolet Photoelectron Spectroscopy (UPS)

The sample was prepared via spin-coating on ITO glass. A He I UV source (hν=21.22 eV) was used and operated at 20 mA. Data was acquired with a pass energy of 5 eV. A 9 V bias was applied to the sample. As photoelectron counts are plotted as a function of binding energy with respect to the Fermi level (E_F), the work function (Φ) and ionization energy (IE) with respect to the vacuum energy level (E_{vac}) can be extracted by the following equations:

$$\Phi = h\nu - (E_{cutoff} - E_F)$$

$$IE = h\nu - (E_{cutoff} - E_{valence})$$

Where E_{cutoff}, and $E_{valence}$ are determined by drawing tangent lines through the cutoff and baseline from secondary electron cutoff (SECO) side and E_F side, respectively.

5.2 Scanning Tunneling Microscopy and Scanning Tunneling Spectroscopy (STM/STS)

All the samples for STM and STS studies were prepared in glove box. The solution for core-only and core-crown NPLs were diluted with octane (ratio 1:1) and deposited by drop-casting 10 μL droplets on a freshly annealed gold (111) surface on mica. Before transferring samples to STM measurements, all samples need enough time to fully get dried in glove box. Both STM imaging and STS measurements of the NPLs were performed on a Bruker Multimode 8 SPM using freshly prepared Pt/Ir (80%:20%) tips at room temperature (19 °C) under ambient conditions. During the approaching process, a current of 1 nA was set at a bias of 0.1 V, which was applied to the sample electrode. During I-V sweeps in STS measurements, the ramp began and ended at 2 and -2 V, respectively. The ramp rate was 4 Hz.

Consecutive I-V sweeps were performed at the center or edge of the same NPLs. For each sweep, the feedback loop was turned off to ensure a fixed tip-sample separation during spectral acquisition. After each sweep, the feedback was re-engaged and a short STM image was acquired to verify that the tip remained positioned on the same nanoplatelet and identifiable morphological features.

5.3 Assumptions in the Comparative Quantification of Trap-Related In-Gap Spectral Intensity

The same comparative formalism can in principle be applied to the full dI/dV spectrum under identical tunneling conditions. In the present work, however, our quantitative analysis is specifically focused on the trap-related in-gap spectral intensity, and the discussion below is therefore written in terms of the relative in-gap contrast. In the standard STS formula, the tunneling current can be written as:

$$I(V) \propto \int \rho_s(E)\rho_t(eV - E)T(E, V, z)dE$$

where ρ_s and ρ_t are the density of states of the sample and tip, respectively, and $T(E, V, z)$ is the

tunneling matrix element, which depends on energy, bias, and tip-sample separation z. (1) Thus, the measured differential conductance is influenced by the sample DOS, tip DOS, and tunneling transmission. The commonly used simplified relation,

$$\frac{dI}{dV} \propto \rho_s(E_F - eV)$$

is employed here as a comparative approximation under identical tunneling conditions, rather than as an absolute DOS formalism.

For the present analysis, the key point is that the trap-state contrast is evaluated by comparing core-only and core-crown spectra at the same bias under the same measurement protocol and with the same Pt/Ir tip. Under these conditions, the tip DOS enters as a common factor in the measured conductance and therefore affects both datasets in the same manner to first order. In this sense, the tip DOS itself is not expected to generate the observed sample-dependent change in in-gap spectral intensity, even though the full tunneling signal is not determined by the sample DOS alone.

To illustrate this point more explicitly, one may define a relative in-gap contrast function as,

$$CF_{gap}(V) = \frac{\left(\frac{dI}{dV}\right)_{core-only} - \left(\frac{dI}{dV}\right)_{core-crown}}{\left(\frac{dI}{dV}\right)_{core-only}}$$

where $(\frac{dI}{dV})_{core-only}$ and $(\frac{dI}{dV})_{core-crown}$ are the measured differential conductance values at the same bias for the core-only and core-crown NPLs, respectively. Under the same first-order approximation used above, the $CF_{gap}(V)$ can be written as,

$$CF_{gap}(V) = \frac{\rho_s^{core-only}(E_F - eV)\rho_t(E_F)T_{eff}^{core-only}(V,z) - \rho_s^{core-crown}(E_F - eV)\rho_t(E_F)T_{eff}^{core-crown}(V,z)}{\rho_s^{core-only}(E_F - eV)\rho_t(E_F)T_{eff}^{core-only}(V,z)}$$

which gives,

$$CF_{gap}(V) = 1 - \frac{\rho_s^{core-crown}(E_F - eV)T_{eff}^{core-crown}(V,z)}{\rho_s^{core-only}(E_F - eV)T_{eff}^{core-only}(V,z)}$$

This equation shows that, for measurements performed at the same bias and the same tip (i.e. Pt/Ir

tip), the tip DOS enters as a common factor and cancels to first order in the relative contrast. The main remaining experimental concern is therefore the possible difference in the effective tunneling transmission $T_{eff}(V,z)$, rather than the tip DOS itself. Under the identical set-point conditions used here, together with the same NPL thickness and ligand system for both core-only and core-crown NPLs, the effective transmission factors are expected to be comparable to first order, i.e.,

$$T_{eff}^{core-crown}(V,z) \approx T_{eff}^{core-only}(V,z)$$

therefore,

$$CF_{gap}(V) = 1 - \frac{\rho_s^{core-crown}(E_F - eV)}{\rho_s^{core-only}(E_F - eV)}$$

Therefore, the relative reduction of in-gap states observed in the selected energy window mainly reflects the difference in sample-related spectral intensity rather than effect from tip DOS and tunneling matrix element. The fixed-window integration of peak-normalized dI/dV spectra used in the manuscript is the integrated form of this comparative contrast analysis, which quantitatively evaluates the difference of trap densities in both NPL systems.

5.4 Tip-induced Band Bending (TIBB) and Screening Effects

To evaluate whether Tip-induced band bending (TIBB) or screening effects could significantly distort the extracted band edges or trap signatures, we used two complementary approaches: (i) SEMITIP electrostatic simulations, (2) and (ii) STS under UV-light illumination. Both analyses indicate that TIBB is present but limited, and is not the dominant origin of the observed band-edge positions or trap-state differences.

To evaluate the possible magnitude of TIBB, we first performed electrostatic simulations using the SEMITIP package. Because most SEMITIP implementations are designed for bulk semiconductor geometries, we used the 'Film2' module, which allows simulation of a thin semiconductor layer on a highly doped semiconductor substrate. This geometry was adopted as an approximate electrostatic model for our ultrathin core-only or core-crown NPL on Au(111), where the metallic substrate provides strong electrostatic screening analogous to a highly conducting support.

As a first step, we considered a simplified electrostatic model consisting of a 1.8 nm thick perovskite layer directly on a highly screened substrate. The purpose of this calculation was not to

reproduce the full microscopic structure of the ligand-coated NPL, but rather to estimate the order of magnitude and parameter dependence of TIBB as a function of tip-sample separation (d), dielectric constant (ε), and surface-state density. The simulation parameters are shown in **Table S9**. Notably, the IEF factor was set as 2, which refers to a scenario that the semiconducting film on top of a semiconducting substrate, and the contact is on the substrate. The module will use the Fermi level of the substrate to calculate the Fermi level of the two films (thin film on substrate) system.

We first examined the effect of tip-sample separation by comparing d = 0.8 nm and d= 0.5 nm under the same dielectric constant (ε = 10) and no surface states conditions, as shown in **Figure S12**. As expected, reducing the separation increases the electric field from the tip and leads to a larger absolute change in the surface potential at the semiconductor surface relative to surface potential at zero sample-tip bias voltage (ΔPot0), corresponding to stronger band bending at the surface. The corresponding electrostatic potential-energy profiles along the z-direction into the semiconductor also show that the smaller separation produces a steeper near-surface potential drop. However, in both cases the potential rapidly converges within the perovskite layer, indicating that the field penetration into the semiconductor remains very limited. This behavior indicates that the electrostatic perturbation is strongly localized near the tip-facing surface. This limited penetration reflects the combined effect of the highly screened substrate, the geometric localization of the tip electric field, and the electrostatic potential division across the layered structure.

We also examined the lateral variation of the potential along the surface. As the radial distance (r-distance) from the tip axis increases, the electrostatic potential decreases, confirming that the perturbation is spatially localized. The lateral extent of the perturbation remains mainly within the characteristic lateral dimensions of the NPLs (~10-15 nm). Since our STS measurements were performed with the tip positioned either at the center or at the edge of the NPL, the most relevant quantity for evaluating the local TIBB is the on-axis potential (r = 0).

We then evaluated the role of dielectric screening by fixing the tip-sample separation at 0.8 nm, no surface states and varying the dielectric constant. The dielectric constant reported for 6-8 nm thick $CsPbBr_3$ nanocrystals is approximately 5.8. (3) We use ε = 5.8 as a reasonable start and ε = 10 as a comparison (**Figure S13.**). Lowering the dielectric constant from 10 to 5.8 produces a larger ΔPot0 and correspondingly stronger electrostatic potential bending along the z-direction,

with near-surface values reaching approximately +0.25 eV at +2 V and -0.17 eV at -2 V. This trend is physically expected from electrostatic potential division: in a layered tip/vacuum/film/substrate geometry, the local potential drop across each region scales approximately with t/ε, where t is the layer thickness and ε is its dielectric constant. In this sense, t/ε represents the effective electrostatic thickness of the layer, i.e., its relative weight in the overall voltage division. Therefore, a smaller dielectric constant causes a larger fraction of the applied field to penetrate into the film. Nevertheless, even in this case the potential still decays rapidly within the 1.8 nm perovskite slab. The lateral potential profiles show the same trend. The perturbation decreases with increasing r, while remaining spatially confined near the tip position. These results indicate that the dielectric constant is an important parameter controlling the magnitude of apparent band bending, while the overall penetration depth remains limited by the combined effects of substrate screening, geometric localization of the tip field, and electrostatic potential division across the layered structure.

We further investigated the effect of surface states by fixing the dielectric constant at 5.8 and the tip-sample separation at 0.8 nm, and comparing three cases: no surface states, an upper-surface state density of 1×10^{12} $cm^{-2}\cdot eV^{-1}$, and an interface-state density of 1×10^{12} $cm^{-2}\cdot eV^{-1}$ at the film/substrate interface (**Figure S14** and **Figure S15**). When surface states are placed at the upper surface, their influence is mainly observed under negative bias. The ΔPot0, the z-dependent potential profile, and the lateral potential profile all show slightly more negative bending, but the additional shift remains limited to only ~0.025 eV even at -2 V. Under positive bias, the curves are nearly unchanged. This behavior can be understood from the fact that the upper-surface states are located at the tip-facing surface, where the local electric field is strongest and where the potential drop is most concentrated. As a result, these states can more effectively modify the local surface charge distribution and thus perturb the near-surface electrostatic potential. The stronger effect under negative bias further suggests that the occupation of these surface states is more readily modulated in that bias direction, leading to a slightly enhanced negative band bending.

In contrast, when the same density of surface states is placed at the film/substrate interface, no significant difference is observed relative to the case without surface states; the calculated ΔPot0, z-dependent potential profiles, and lateral potential profiles essentially overlap. This is consistent with the combined electrostatic picture: by the time the tip-induced electric field reaches the buried

interface, it has already decayed substantially due to near-surface field localization and layered potential division, and any remaining perturbation is further efficiently screened by the substrate. Therefore, interface states located at the film/substrate interface have only a very limited ability to modulate the tip-induced electrostatics.

The simplified model shows that the magnitude of TIBB is much more sensitive to the tip-sample separation and dielectric constant, while the influence of surface states is more modest and bias-dependent. However, the parameter sets yielding the largest calculated band bending would also lead to an apparent STS bandgap substantially smaller than the independently measured optical bandgap from UV-vis absorption (corresponding to an apparent bandgap narrowing of ~0.3 eV), indicating that these extreme assumptions overestimate the electric-field penetration into the NPL. This discrepancy is expected because the simplified model neglects the ligand layers present on both the top and bottom surfaces of the NPLs. In the actual samples, these ligand shells provide additional dielectric spacing and electrostatic screening, which reduce the fraction of the potential drop occurring inside the perovskite layer.

To account for this effect, we constructed a more realistic effective model in which the ligand-coated structure is represented by an increased electrostatic thickness. Using a series-capacitor approximation, the 1.8 nm perovskite slab was combined with top and bottom ligand layers to estimate the effective potential division across the full NPL structure. The effective thin film thickness (t_{eq}) is given by:

$$t_{eq} = t_p + \frac{\varepsilon_p}{\varepsilon_L} (t_{L,top} + t_{L,bottom})$$

Where t_p, $t_{L,top}$, and $t_{L,bottom}$ are the thickness of perovskite slab, top ligand layer and bottom ligand layer, respectively. ε_p and ε_L are the dielectric constant of perovskite layer and ligand layer. We set $\varepsilon_L = 2.5$, based on the reported dielectric constant of oleic acid. The full length of the ligand is 1.5 nm, (4-6) considering the measurements were performed in air and stacking effect, we adopted an effective thickness of both top and bottom ligand as 0.5 nm. This yields an effective total thin film thickness of 4.12 nm. The simulation parameters are shown in **Table S10**.

In this ligand-inclusive model, as shown in **Figure S16**, the calculated ΔPot0 at the tip-facing outer surface reaches approximately ±0.6 eV at ±2 V, which is larger than in the simplified model.

However, this larger surface potential shift occurs mainly within the ligand region and does not imply stronger band bending inside the perovskite. Once the electrostatic potential is evaluated at the equivalent top surface of the perovskite layer (located ~1.16 nm below the outer ligand surface), the electrostatic potential energy is reduced to only at ~0.07 eV at +2 V and ~-0.03 eV at -2 V (**Figure S16c**). The potential then decays rapidly and converges to nearly zero within ~1.5-1.6 nm below the tip-facing surface (~0.5-0.6 nm within the perovskite layer). This behavior indicates that the tip-induced perturbation remains strongly localized near the surface and that the ligand shell primarily redistributes the potential drop rather than extending the spatial range of band bending.

Based on these values, the TIBB-induced shift of the experimentally relevant $CsPbBr_3$ and $FAPbBr_3$ band edges is estimated to be only on the order of ~-0.02 eV for the VBM and ~0.05-0.06 eV for the CBM. These values are substantially smaller than the experimentally observed core-crown band-edge separation (~0.12-0.14 eV) and are insufficient to account for the deep-trap spectral intensity and progressive gap-filling behavior. As shown in **Figure S17a** and **Figure S17b**, the VB and CB positions for all location categories (e.g., core-only center, core-crown center and core-crown edge) mainly retain the same. Therefore, although a small amount of TIBB cannot be completely excluded, both the illumination test and the SEMITIP analysis indicate that TIBB is not the dominant factor of the observed band-edge positions, in-gap trap signatures, or the pronounced differences between the core-only and core-crown NPLs.

In addition, we performed STS measurements at the edge of core-crown NPLs under UV illumination as an experimental test of the possible contribution of TIBB. All sample preparation procedures and experimental parameters are same as the parameters described in **section 5.2**. Previous light-modulated STM/STS studies on halide perovskites have shown that optical excitation can modify the local electrostatic environment sensed by the tunneling junction by generating photocarriers, changing carrier distributions, and inducing surface-photovoltage-related effects. In particular, light-modulated STM has been used to visualize photocarrier generation at perovskite heterointerfaces, and illumination-dependent STM/STS measurements have been shown to alter the local band alignment and electric field distribution in perovskite systems. These studies establish that illumination can serve as a useful experimental perturbation for evaluating whether junction electrostatics, including TIBB, strongly influence the measured

spectra. (7-9)

Unlike many previously studied iodide-rich or mixed-halide perovskites, for which visible excitation such as 532 nm is already above the bandgap, the strongly confined $CsPbBr_3$/$FAPbBr_3$ NPLs investigated here exhibit a substantially wider optical gap (~2.7-2.8 eV). Therefore, UV illumination was used in our measurements to ensure direct inter-band photoexcitation and efficient photocarrier generation in this wide-bandgap system. If TIBB were a dominant factor in our measurements, one would expect noticeable changes in the apparent band-edge positions and/or major spectral features under illumination. In our measurement (as shown in **Figure S18**), the dI/dV spectra at the edge of core-crown NPLs acquired under UV illumination show VBM/CBM values within the same band-edge range and retain the same principal VB/CB peak features as those measured without illumination. This observation suggests that, under our experimental conditions, TIBB is limited and does not dominate the extracted band edges or the observed trap-state signatures.

Meanwhile, we note that UV illumination can perturb halide perovskites not only through near-surface electrostatics but also through photoinduced surface chemical changes, such as depassivation and accelerated degradation. (10) Accordingly, the UV-assisted STS measurements were intentionally limited to short illumination times and used only as a qualitative perturbation test rather than as a prolonged illumination experiment. During data analysis, we focused on spectra acquired before the onset of obvious irreversible degradation and excluded traces showing clear bandgap-closure-like behavior analogous to that observed in consecutive I-V sweeps measurement. We therefore interpret the UV-assisted STS experiment as supportive evidence that TIBB is not the dominant origin of the observed spectral differences, rather than as a stand-alone quantitative measure of TIBB.

6. Theoretical Calculations

All theoretical calculations were carried out in the Vienna Ab-initio Simulation Package (11-15) with the projector-augmented-wave method. The orthorhombic phase of $CsPbBr_3$ was considered. To create the FA-substituted system, the unit cell was first relaxed (ISIF = 3) and then one of the Cs atoms was replaced with an FA molecule. This results in a system with 75% Cs and 25% FA. Another system with 100% FA substitution was created for comparative analysis. Both substituted systems were further relaxed for determining electronic structure of these systems. Additionally,

the orthorhombic $FAPbBr_3$ was considered to contrast with the 100% substituted system (starting with the $CsPbBr_3$ system). These systems are presented in **Figure S19**. The Perdew-Burke-Ernzerhof (PBE) functional was used for relaxing geometry. All calculations were spin-polarized. The PDOS and the band structures were calculated with the Heyd-Scuseria-Ernzerhof 2006 (HSE06) functional (16), which employs 25% Hartree-Fock exact exchange. Both the PBE and HSE06 functionals included Grimme's D3 corrections (17-18). The plane wave basis kinetic energy cutoff used was 500 eV, with convergence criteria for the self-consistent field (SCF) set to 10^{-6} eV for the energies and 0.02 eV Å^{-1} for the residual forces. The band structure calculations used an increased SCF convergence of 10^{-8} eV. A k-point grid of 3 x 3 x 2 was employed for all systems. Monkhorst-Pack was used to generate the k-point grids (19). To create the charge density difference plots, the first triplet excited states were probed using the Delta-SCF method (10). Post processing of the VASP data for the PDOS and band structure data was done with VASPKIT and the sumo python package, respectively (21-22). The charge density difference plots and system snapshots were visualized using VESTA (23).

7. Supplementary Tables

Table S1. Quantification table determined from XPS for core NPLs.

	BE (eV)	FWHM (eV)	RSF	Atomic Concentration (%)
Pb 4f A	136.44	1.11	8.33	0.2
Pb 4f B	138.24	0.93	8.33	1.6
O 1s	532.15	0.00	0.78	20.2
N 1s	401.65	0.00	0.48	1.2
In 3d	444.05	0.00	7.27	3.4
Cs 3d	724.45	0.00	11.74	1.3
Br 3d	68.15	0.00	1.06	4.5
C 1s	284.55	0.00	0.28	67.6

Table S2. Quantification table determined from XPS for core-crown NPLs.

	BE (eV)	FWHM (eV)	RSF	Atomic Concentration (%)
Pb 4f A	136.50	1.15	8.33	0.1
Pb 4f B	138.23	0.93	8.33	1.4
O 1s	532.18	0.00	0.78	19.0
N 1s	401.58	0.00	0.48	1.4
In 3d	443.88	0.00	7.27	2.2
Cs 3d	724.38	0.00	11.74	0.9
Br 3d	68.18	0.00	1.06	3.9
C 1s	284.58	0.00	0.28	71.3

Table S3. Comparison of the emission peak energy, Stokes shift and full width at half maximum (FWHM) of core-only and core-crown NPLs.

Sample	Emission (eV)	Stokes shift (eV)	FWHM (eV)
Core-only	2.70	0.09	0.10
Core-crown	2.76	0.05	0.15

Table S4. The best-fit parameters for PL decay dynamics of NPLs.

Sample	τ_1 (ns)	A_1 (%)	τ_2 (ns)	A_2 (%)	τ_3 (ns)	A_3 (%)	τ_{avg} (ns)
Core-Crown	1.85	42.4	4.77	19.95	0.53	37.65	3.15
Core	1.42	44.02	3.80	20.57	0.40	35.41	2.52

Table S5. Histogram-derived band-edge positions for different measurement location categories.

Sample	VBM (eV)	CBM (eV)	Eg (eV)
Core-only center	-1.12	1.56	2.68
Core-crown center	-1.15	1.58	2.73

Core-crown edge 1 (core-like)	-1.14	1.53	2.67
Core-crown edge 2 (crown-like)	-1.28	1.65	2.93

Table S6. Histogram-derived band-edge positions for the center of core-only NPLs across different Pt/Ir STM tips.

Tip number	VBM (eV)	CBM (eV)
#1	-1.10	1.57
#2	-1.13	1.56
#3	-1.13	1.56

Table S7. Histogram-derived band-edge positions for the center of core-crown NPLs across different Pt/Ir STM tips.

Tip number	VBM (eV)	CBM (eV)
#1	-1.14	1.55
#2	-1.15	1.59

Table S8. Histogram-derived band-edge positions for the edge of core-crown NPLs across different Pt/Ir STM tips.

Tip number	VBM (eV)		CBM (eV)	
	Core-like	Crown-like	Core-like	Crown-like
#1	-1.10	-1.22	1.53	1.65
#2	-1.12	-1.25	1.55	1.66
#3	-1.14	-1.27	1.57	1.68

Table S9. Simplified SEMITIP model parameters.

Parameters	Value

Tip-sample separation (nm)	0.5, 0.8
Tip radius (nm)	20
Thickness of the film (nm)	1.8
Region to use in computing Fermi level (IEF)	2
Bandgap of thin film (eV)	2.79
Bandgap of the substrate (eV)	0.5
Donor concentration (cm^{-3})	5.9×10^{22}
Dielectric constant in thin film	5.8, 10
Dielectric constant in substrate	6.9
Temperature (K)	292
Density of first distribution of surface states (surface) ($cm^{-2}\cdot eV^{-1}$)	0, 1×10^{12}
Density of first distribution of surface states (interface) ($cm^{-2}\cdot eV^{-1}$)	0, 1×10^{12}

Table S10. Ligand-inclusive effective SEMITIP model parameters.

Parameters	Value
Tip-sample separation (nm)	0.8
Tip radius (nm)	20
Thickness of the film (nm)	4.12
Region to use in computing Fermi level (IEF)	2
Bandgap of thin film (eV)	2.79
Bandgap of the substrate (eV)	0.5
Donor concentration (cm^{-3})	5.9×10^{22}
Dielectric constant in thin film	5.8
Dielectric constant in substrate	6.9
Temperature (K)	292
Density of first distribution of surface states (surface) ($cm^{-2}\cdot eV^{-1}$)	0
Density of first distribution of surface states (interface) ($cm^{-2}\cdot eV^{-1}$)	0

Table S11. Summary of cell dimensions after relaxation used in theoretical calculations with percent (%) difference from experimental values reported in parentheses.

Compound	a (Å)	b (Å)	c (Å)	α (°)	β (°)	γ (°)
$CsPbBr_3$ (0% FA)	8.296 (2.38)	8.300 (1.84)	11.925 (0.39)	90 (0.00)	90 (0.00)	90 (0.00)
25% FA	8.002	8.724	11.966	89.95	89.65	89.44
100% FA (a-aligned)	9.034	7.842	12.110	90	90	90
100% FA (b-aligned)	7.792	9.097	12.149	90	90	90
$FAPbBr_3$	9.117 (7.35)	7.850 (7.61)	12.068 (0.73)	89.99 (0.01)	90 (0.00)	90 (0.00)

Table S12. Summary of the calculated band gaps, effective masses, and reduced effective masses. All band gaps are centered on the Γ-point.

Systems Functional	Bandgap (eV) HSE06+D3	Direction of effective mass from Γ-point	Effective Mass (in units of m_0)		Reduced Effective Mass
			m_e^*	m_h^*	$\mu = \frac{m_e^* m_h^*}{(m_e^* + m_h^*)}$
$CsPbBr_3$ (0% FA)	2.554	Z	0.103	0.206	0.069
		X	1.032	0.234	0.191
		Y	1.074	0.220	0.183
25% FA	2.595	Z	1.146	0.211	0.178
		X	0.270	0.236	0.126
		Y	0.220	0.265	0.120

100% FA (a-aligned)	2.601	Z	0.869	0.215	0.172
		X	0.295	0.295	0.148
		Y	0.240	0.217	0.114
100% FA (b-aligned)	2.679	Z	0.901	0.226	0.181
		X	0.239	0.232	0.118
		Y	1.290	0.332	0.264
$FAPbBr_3$	2.392	Z	0.840	0.194	0.158
		X	0.205	0.240	0.111
		Y	0.203	0.159	0.089

8. Supplementary Figures

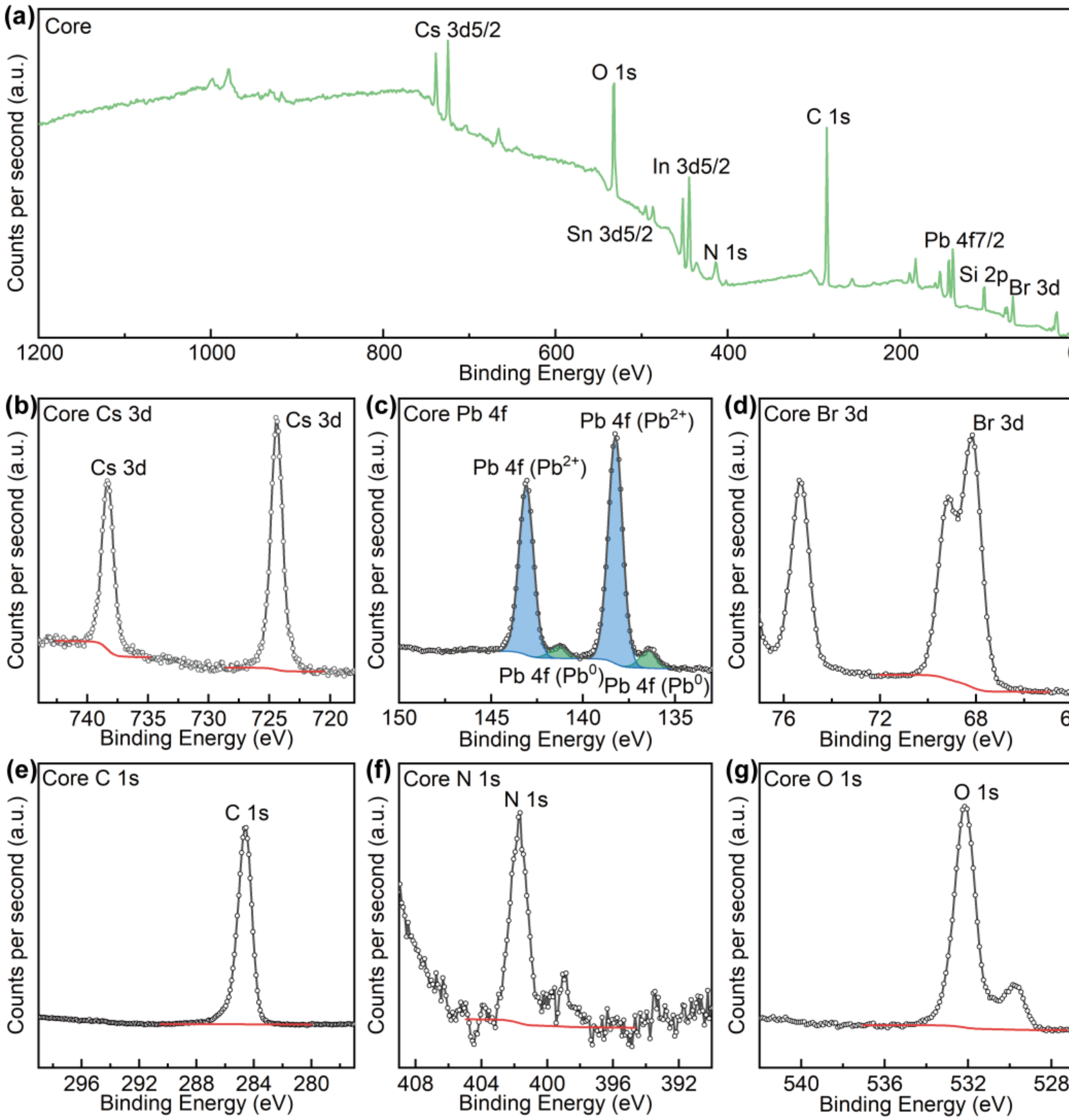

Figure S1. XPS spectra of core NPLs. (a) XPS wide-scan spectrum. (b) XPS high resolution spectrum of Cs 3d. (c) XPS high resolution spectrum of Pb 4f. (d) XPS high resolution spectrum of Br 3d. (e) XPS high resolution spectrum of C 1s. (f) XPS high resolution spectrum of N 1s. (g) XPS high resolution spectrum of O 1s.

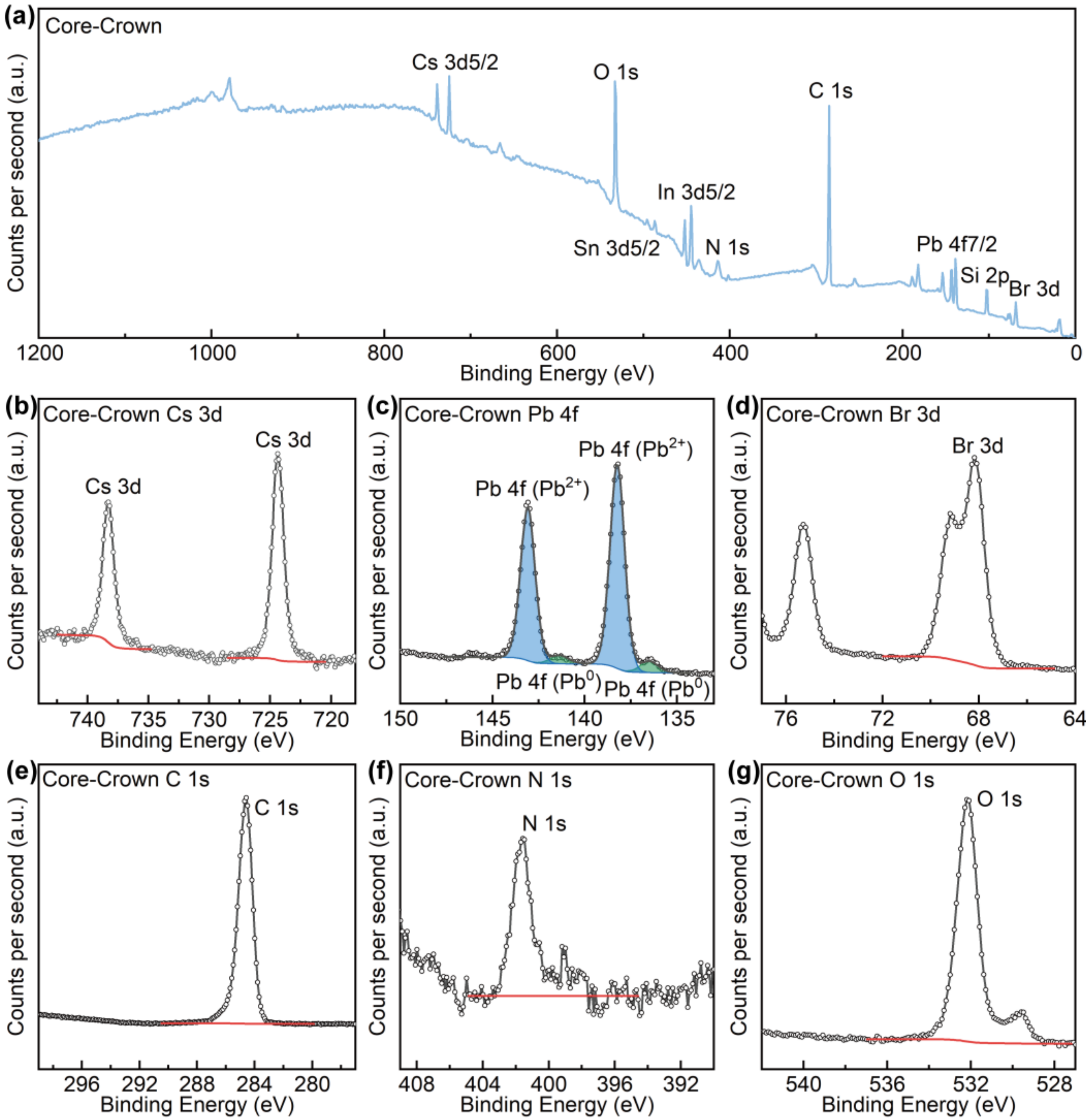

Figure S2. XPS spectra of core-crown NPLs. (a) XPS wide-scan spectrum. (b) XPS high resolution spectrum of Cs 3d. (c) XPS high resolution spectrum of Pb 4f. (d) XPS high resolution spectrum of Br 3d. (e) XPS high resolution spectrum of C 1s. (f) XPS high resolution spectrum of N 1s. (g) XPS high resolution spectrum of O 1s.

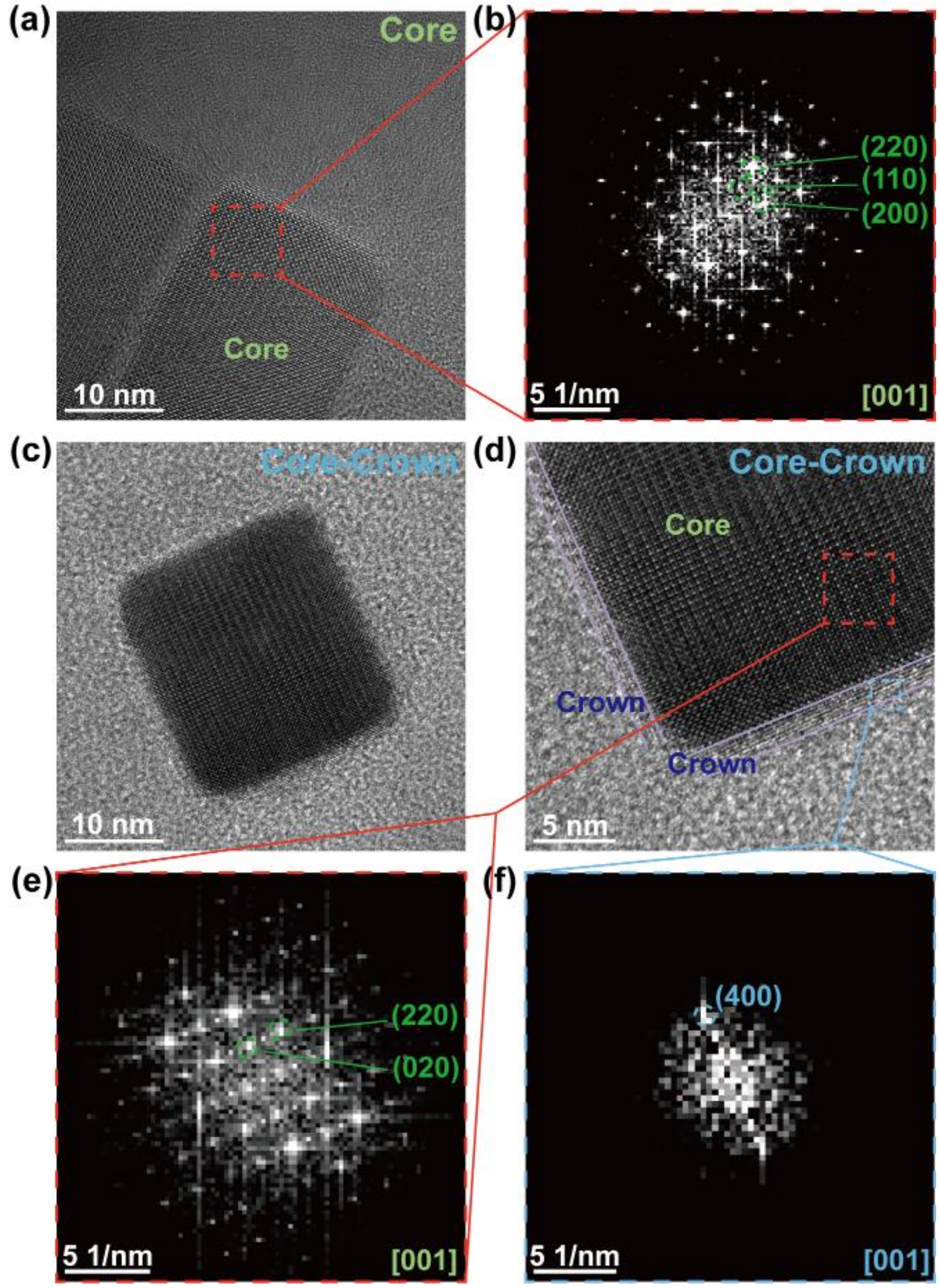

Figure S3. Microstructures of core and core-crown NPLs. (a) HRTEM image of core NPLs and (b) corresponding fast Fourier transform (FFT) pattern of the area in red ROI in (a). (c-d) HRTEM images of core-crown NPLs and corresponding FFT patterns of (e) core and (f) crown regions in red and blue ROIs in (d), respectively.

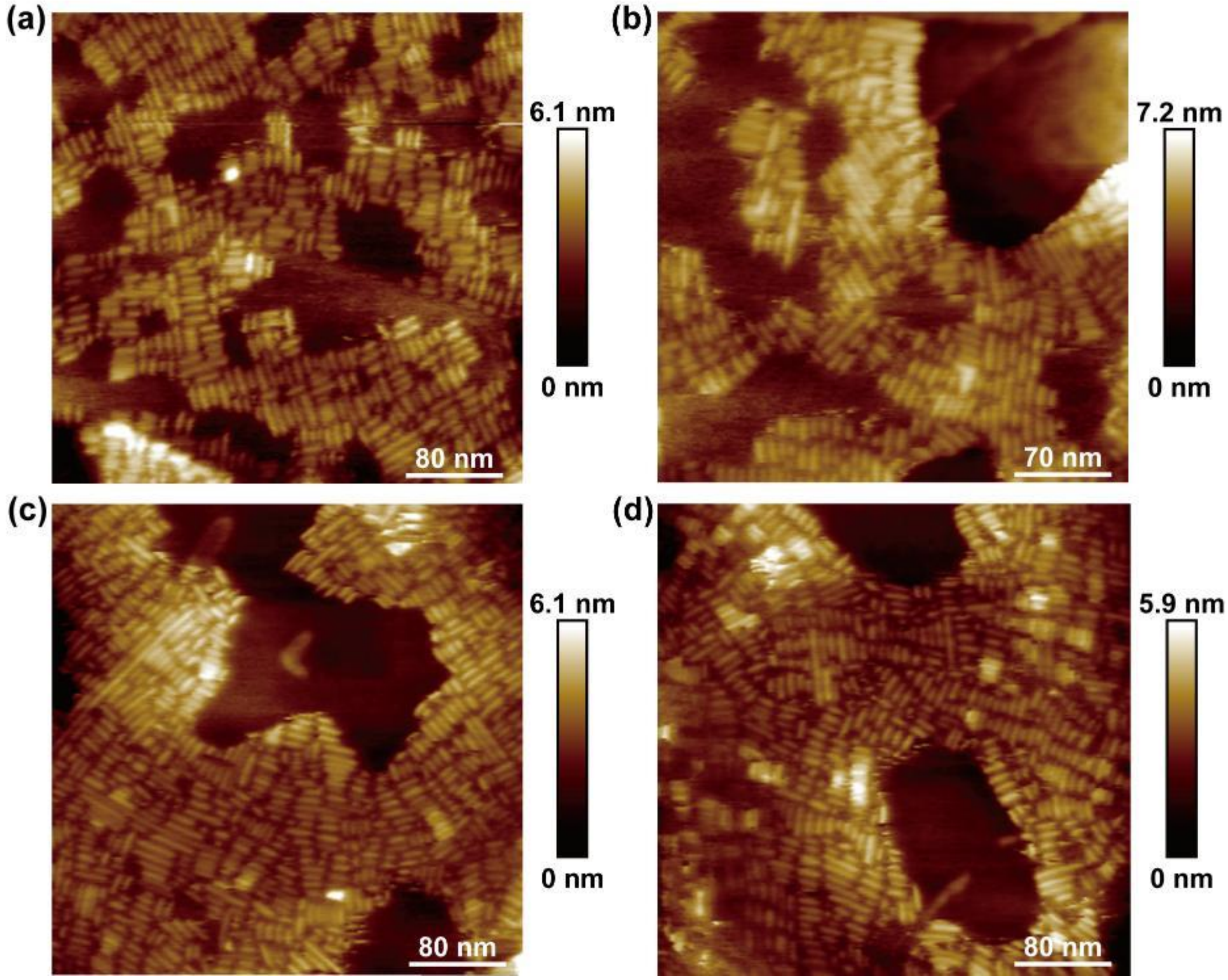

Figure S4. AFM imaging of (a-b) core and (c-d) core-crown NPLs on Au(111) surface.

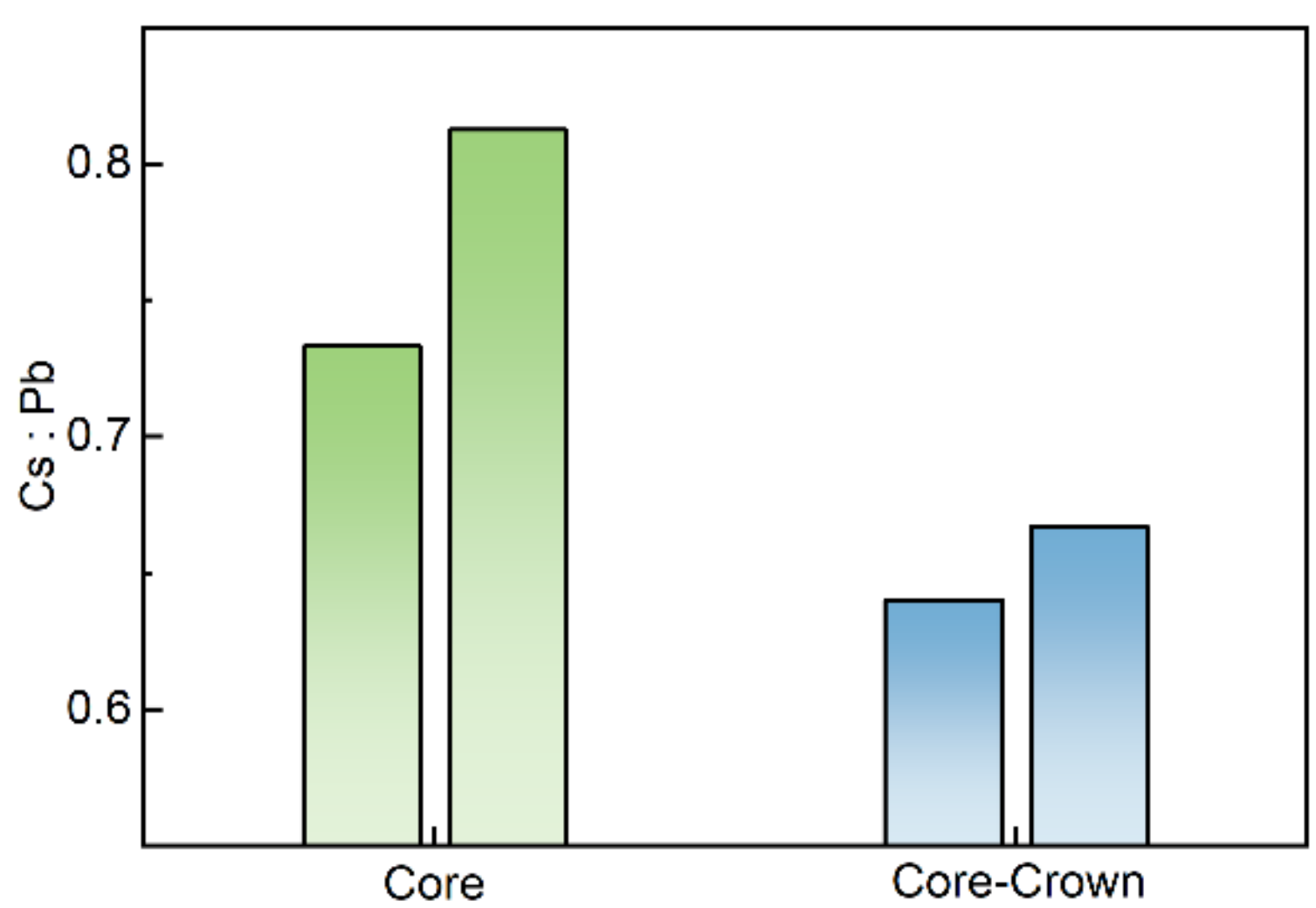

Figure S5. Cs:Pb atomic concentration ratio comparison of core and core-crown NPLs from XPS.

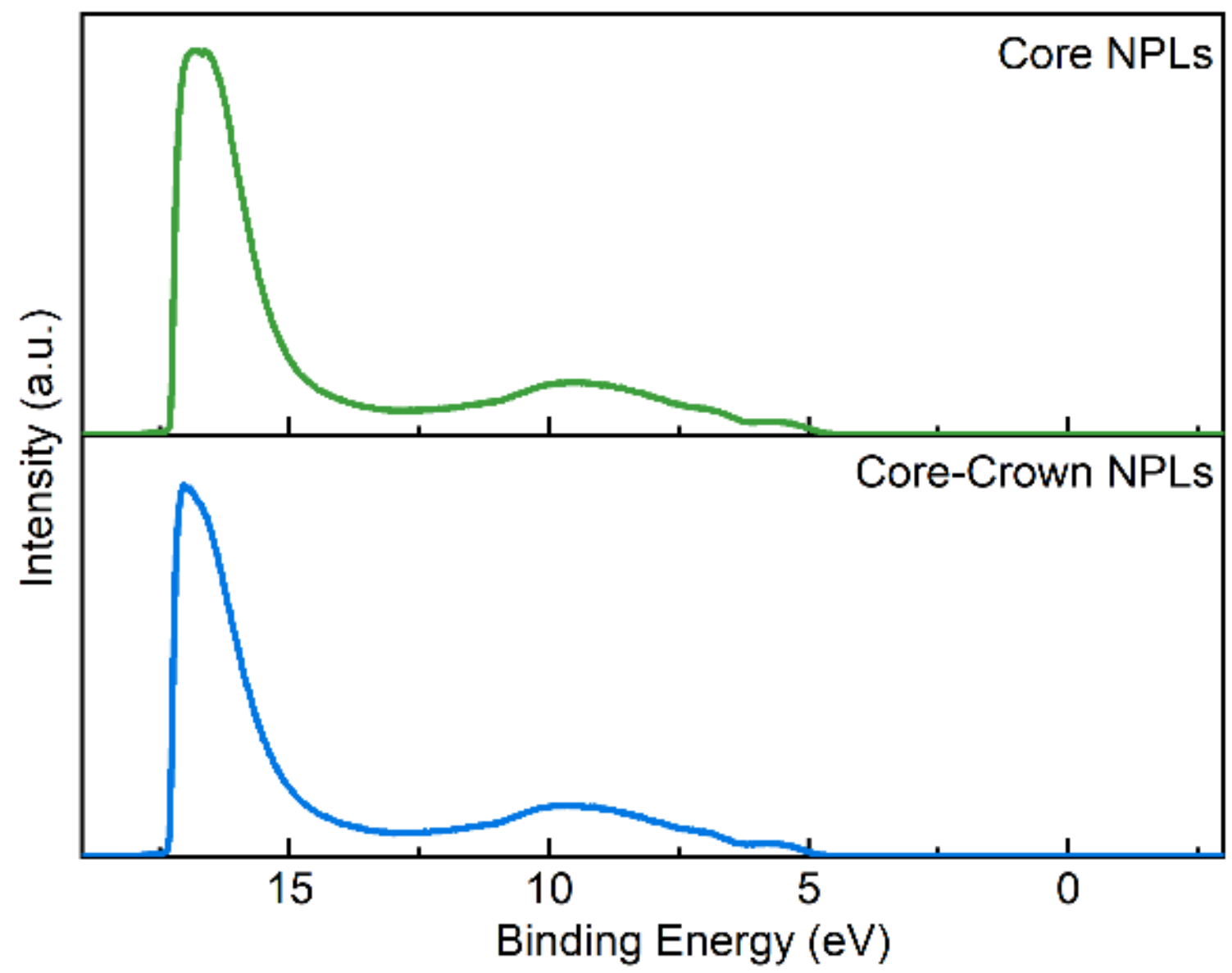

Figure S6. UPS wide-scan spectra for core and core-crown NPLs.

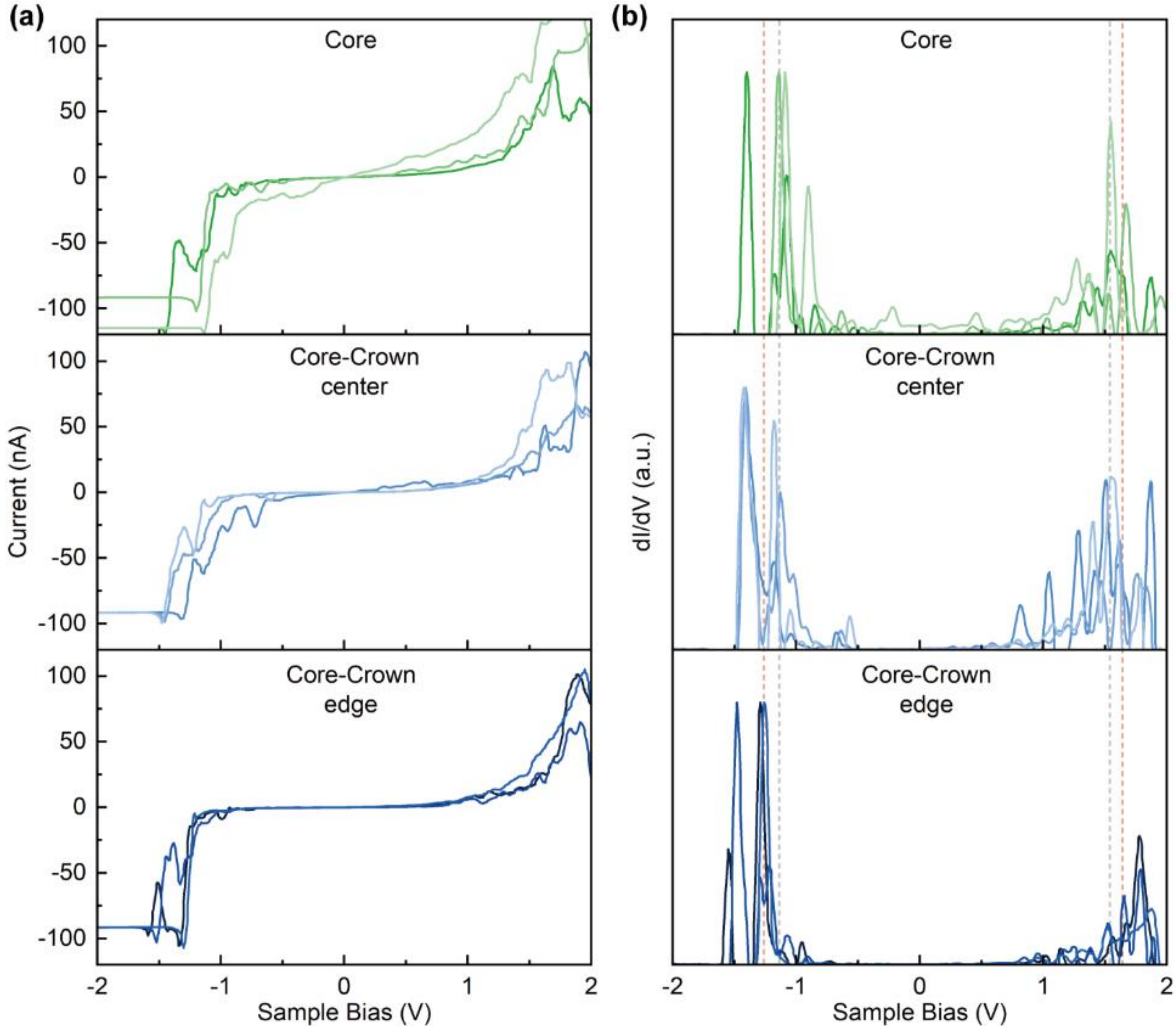

Figure S7. Representative STS (a) I-V sweep traces and (b) the corresponding dI/dV spectra of core-only NPLs and both center and edge of core-crown NPLs. The gray and orange dashed lines represent the VBM and CBM positions of $CsPbBr_3$ and $FAPbBr_3$, respectively.

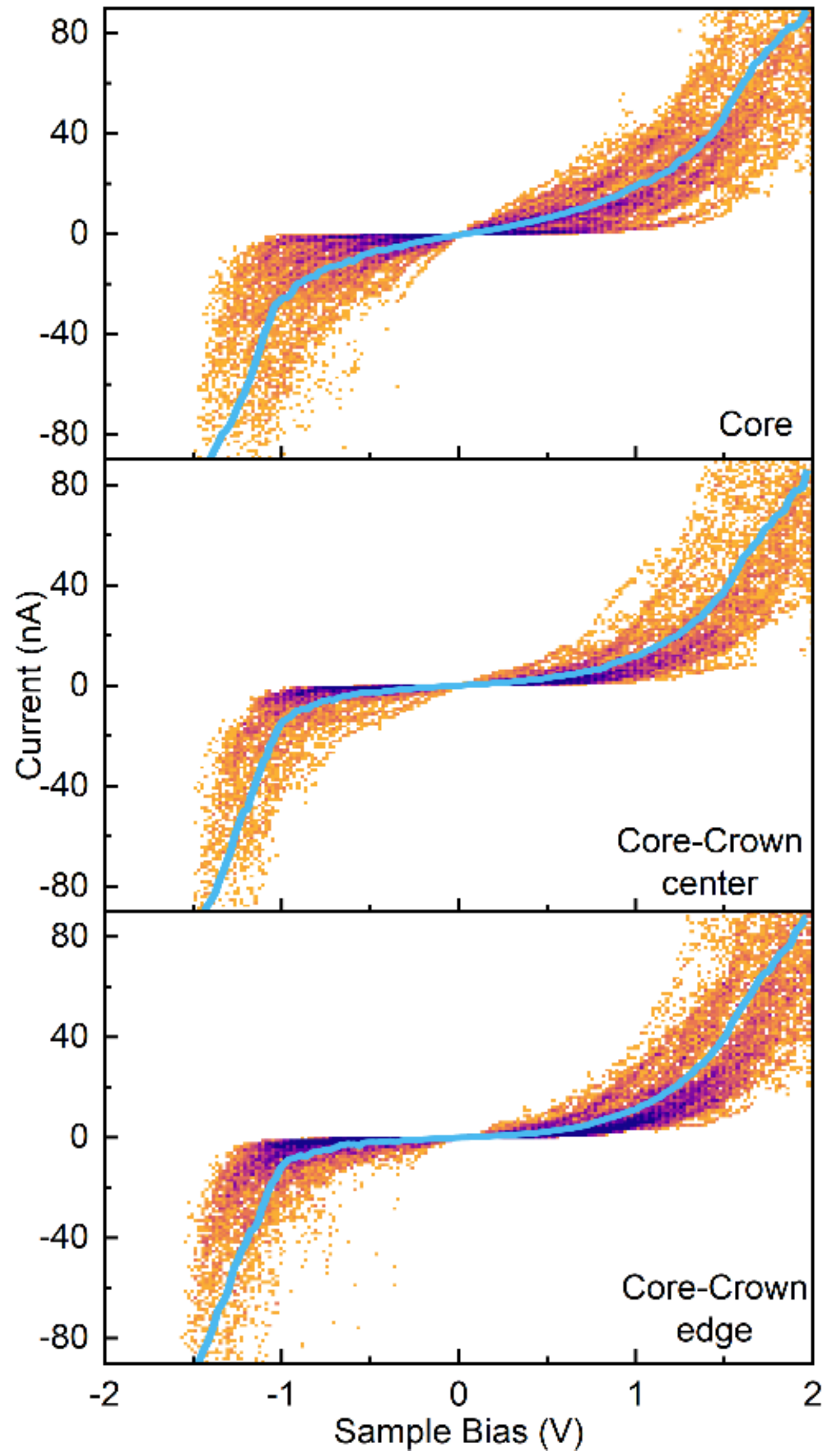

Figure S8. STS I-V map of core NPLs and core-crown NPLs when the STM tip was positioned at the center of both core and core-crown NPLs, as well as at the edge of the core-crown NPLs. The blue curves represent the average of I-V traces.

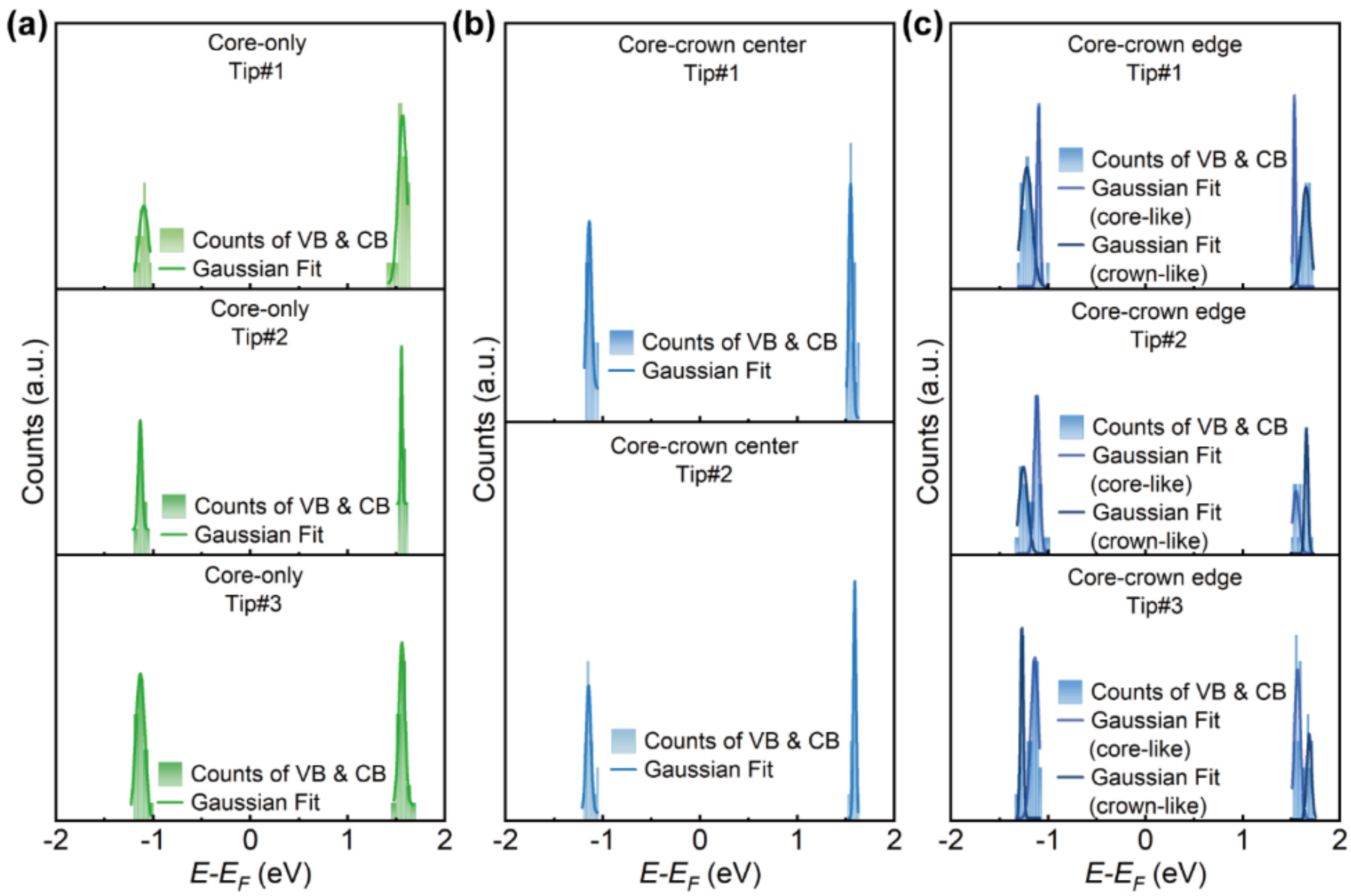

Figure S9. Histograms of VB and CB across different independent tips for (a) center of core NPLs, (b) center of core-crown NPLs and (c) edge of-1. core-crown NPLs.

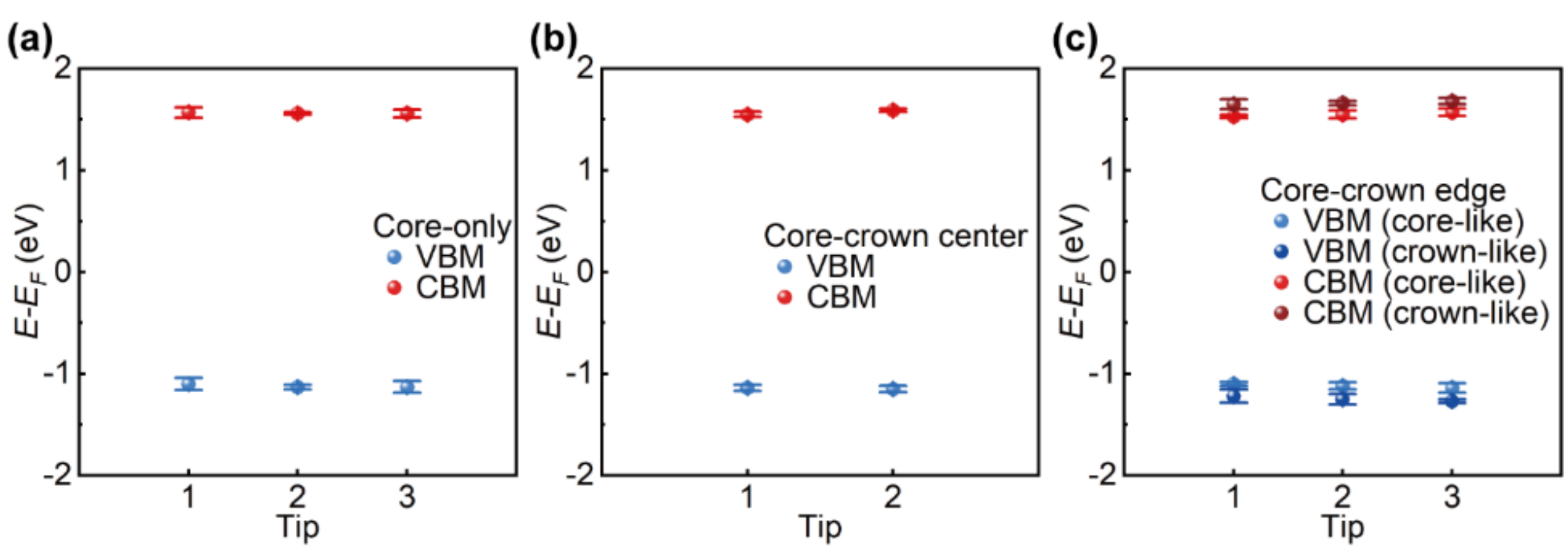

Figure S10. Histogram-derived peak positions of the VBM and CBM for (a) the center of core-only, (b) the center of core-crown and (c) the edge of core-crown NPLs measured using 3, 2, and 3 independently prepared Pt/Ir STM tips. Error bars represent the full width at half maximum (FWHM) of the corresponding histogram peaks.

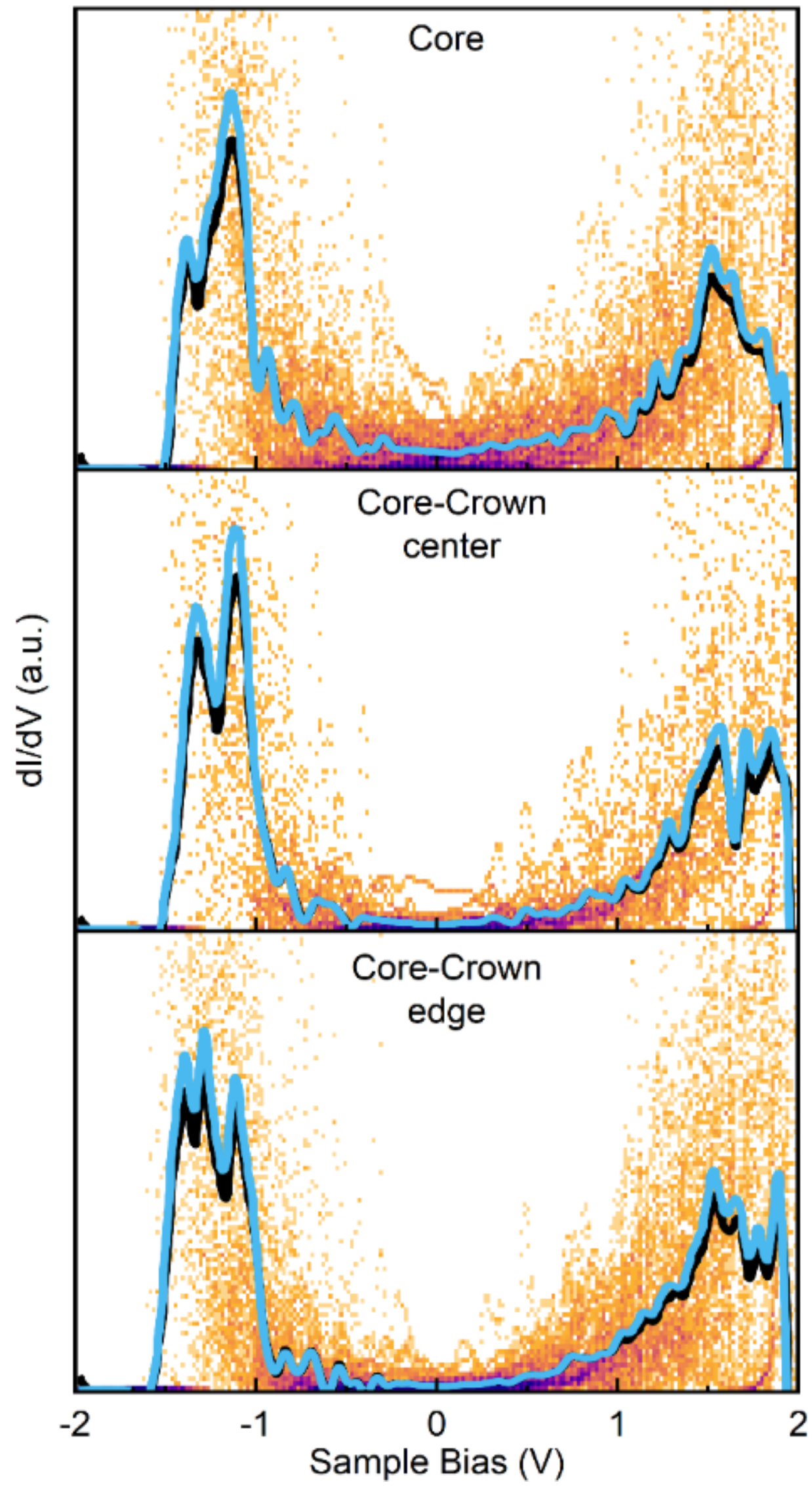

Figure S11. STS dI/dV map of core NPLs and core-crown NPLs when the STM tip was positioned at the center of both core and core-crown NPLs, as well as at the edge of the core-crown NPLs. The black curve is obtained by differentiating the average I-V curve, whereas the blue curve is obtained by averaging the individually differentiated dI/dV spectra.

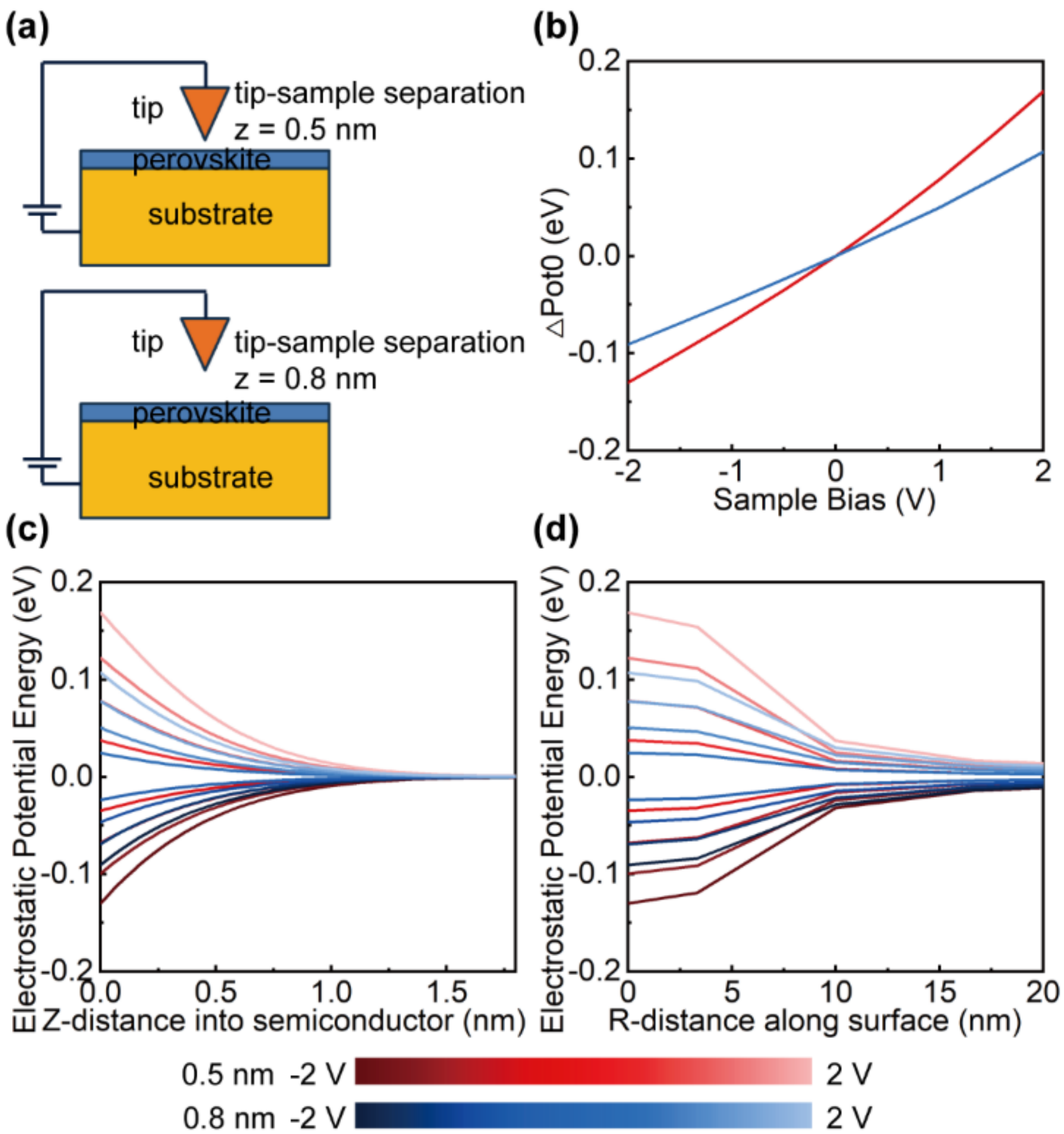

Figure S12. (a) Schematic illustration of the simplified model used in the SEMITIP simulations. This model is used to evaluate how the tip-sample separation (z) affects the electrostatic response of the junction, in particular the surface-potential shift ΔPot0. (b) Surface-potential shift relative to the zero-bias condition, ΔPot0, as a function of sample bias. (c) Electrostatic potential energy profile along the z direction within the perovskite layer at r = 0. (d) Electrostatic potential energy profile along the lateral direction on the top surface (z = 0) of the perovskite layer. The color palettes show the orientation of tip-sample bias voltage change from -2 V to 2 V. The red and blue curves represent the electrostatic response under z = 0.5 nm and z = 0.8 nm, respectively.

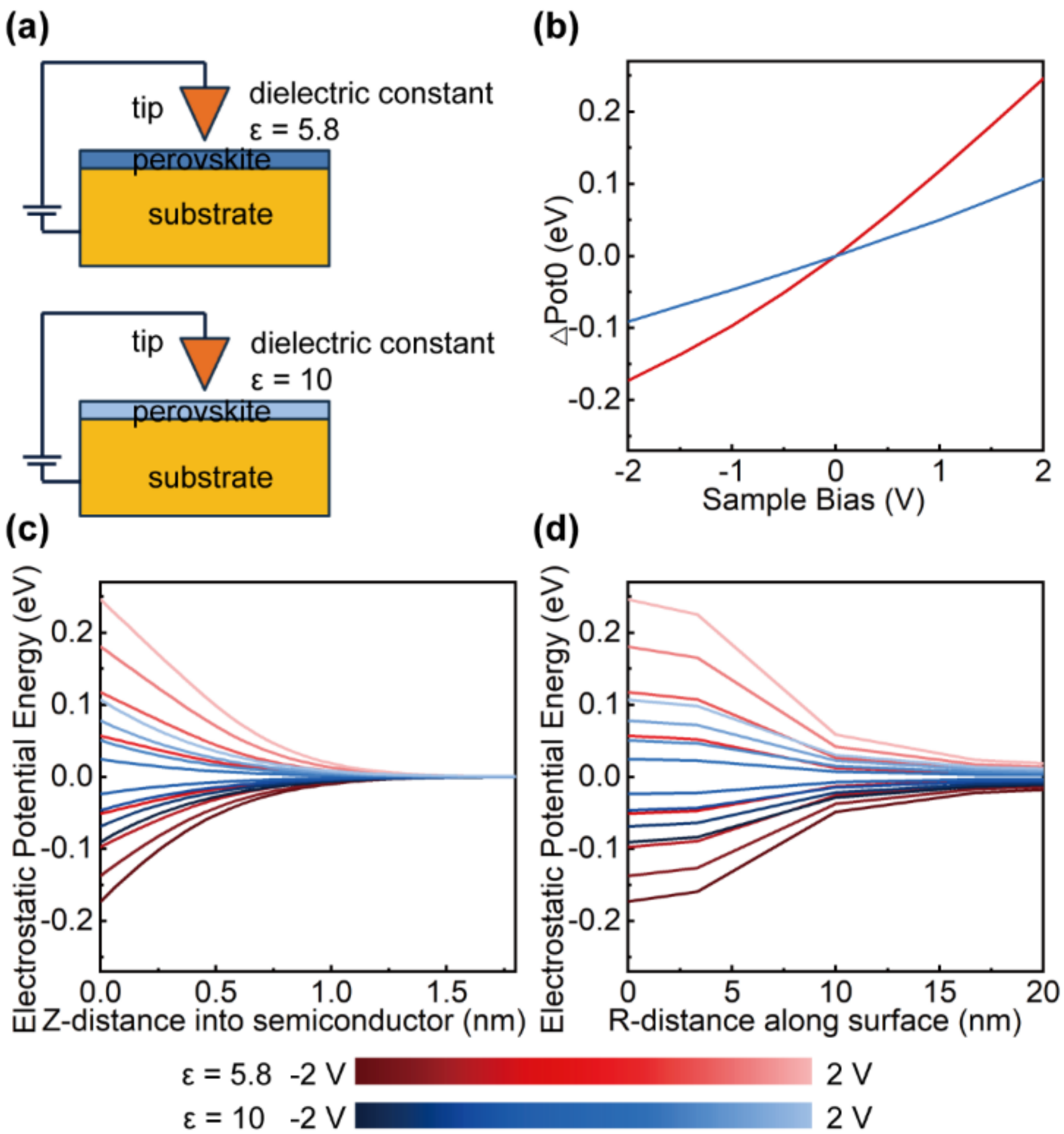

Figure S13. (a) Schematic illustration of the simplified model used in the SEMITIP simulations. This model is used to evaluate how the dielectric constant (ε) affects the electrostatic response of the junction, in particular the surface-potential shift ΔPot0. (b) Surface-potential shift relative to the zero-bias condition, ΔPot0, as a function of sample bias. (c) Electrostatic potential energy profile along the z direction within the perovskite layer at r = 0. (d) Electrostatic potential energy profile along the lateral direction on the top surface (z = 0) of the perovskite layer. The color palettes show the orientation of tip-sample bias voltage change from -2 V to 2 V. The red and blue curves represent the electrostatic response under ε = 5.8 and ε = 10, respectively.

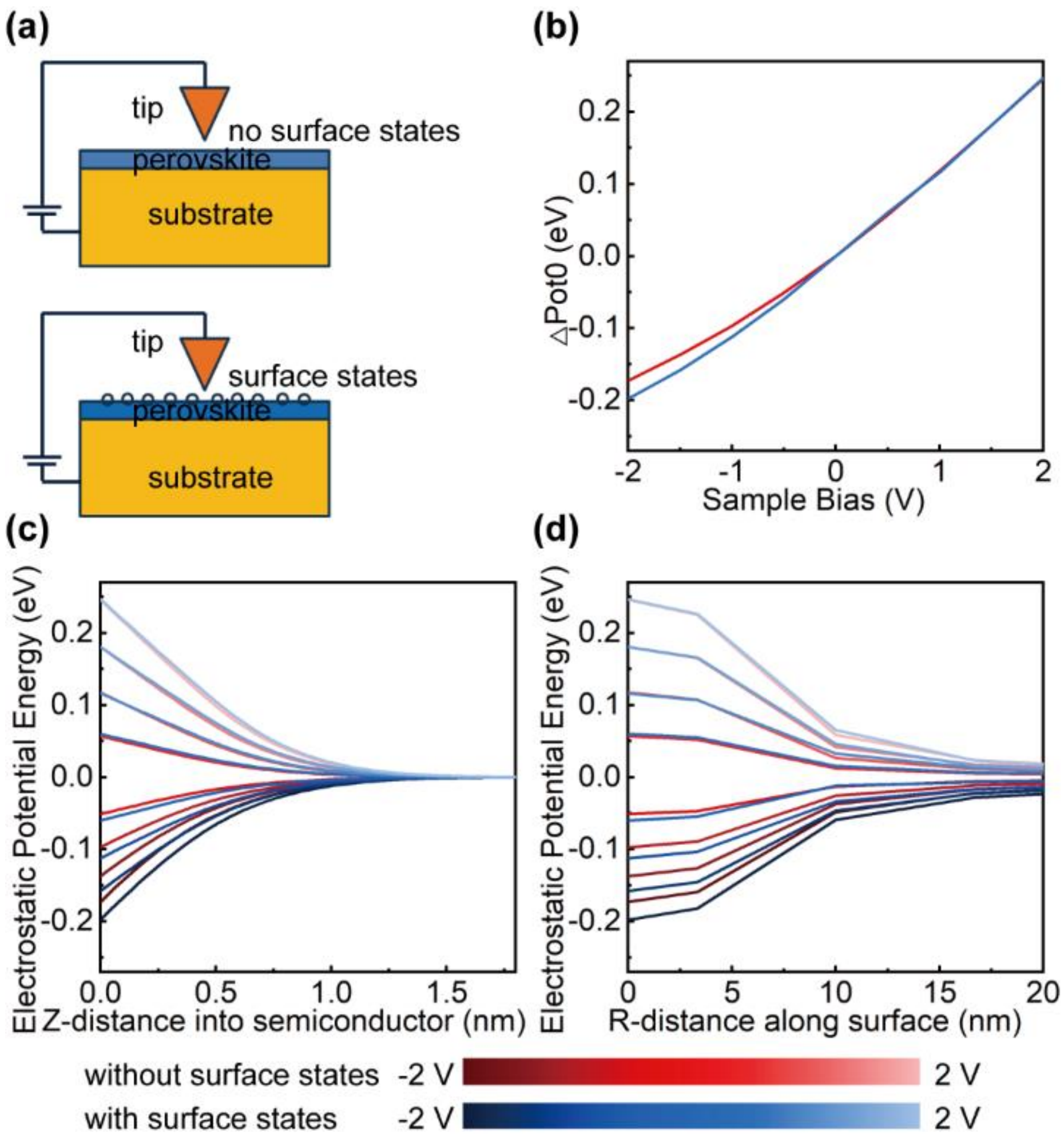

Figure S14. (a) Schematic illustration of the simplified model used in the SEMITIP simulations. This model is used to evaluate how the surface states on the top surface of perovskite film affects the electrostatic response of the junction, in particular the surface-potential shift ΔPot0. (b) Surface-potential shift relative to the zero-bias condition, ΔPot0, as a function of sample bias. (c) Electrostatic potential energy profile along the z direction within the perovskite layer at r = 0. (d) Electrostatic potential energy profile along the lateral direction on the top surface (z = 0) of the perovskite layer. The color palettes show the orientation of tip-sample bias voltage change from -2 V to 2 V. The red and blue curves represent the electrostatic response with and without surface states on the top surface of perovskite film, respectively.

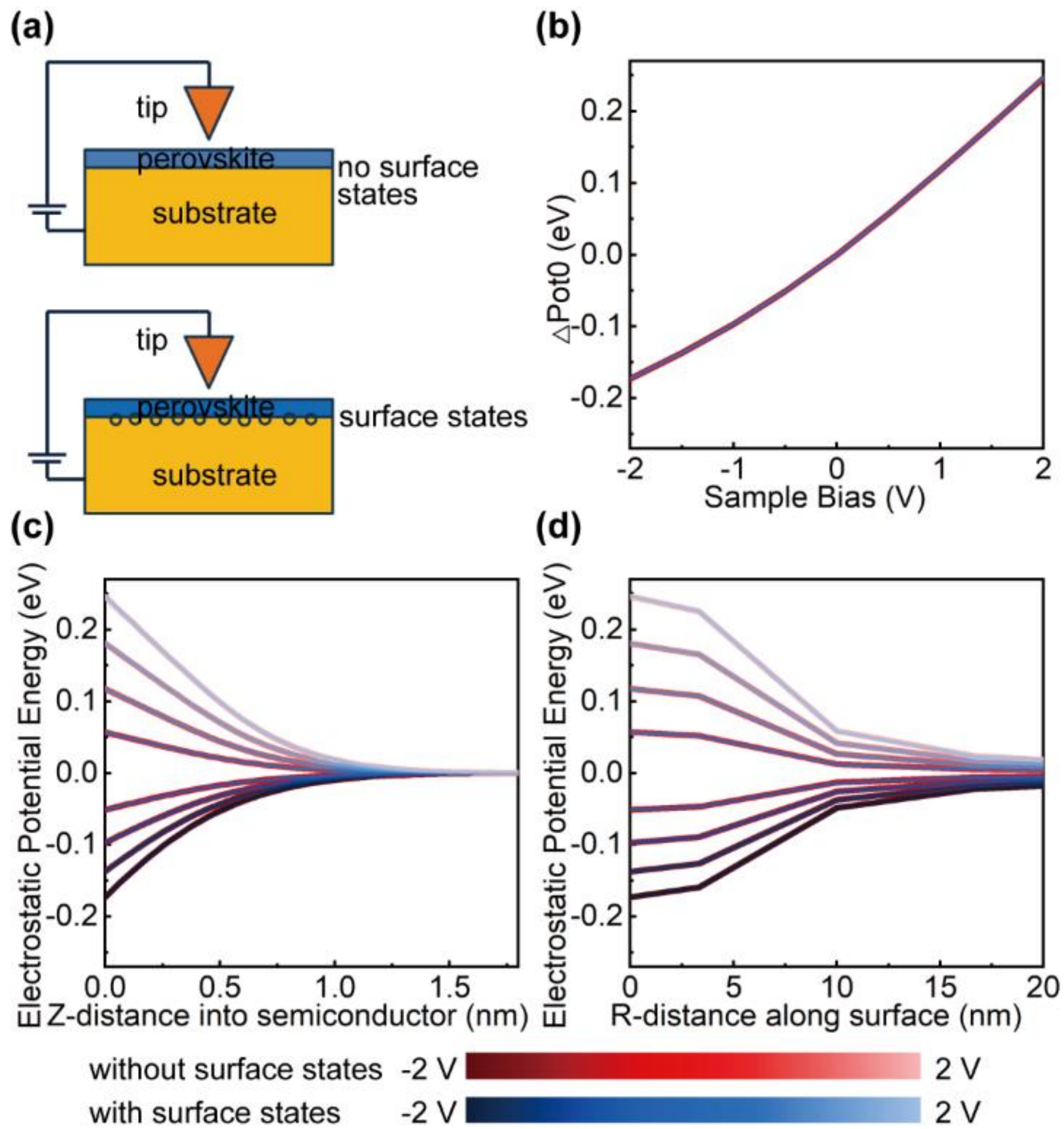

Figure S15. (a) Schematic illustration of the simplified model used in the SEMITIP simulations. This model is used to evaluate how the surface states at the bottom surface of perovskite film affects the electrostatic response of the junction, in particular the surface-potential shift ΔPot0. (b) Surface-potential shift relative to the zero-bias condition, ΔPot0, as a function of sample bias. (c) Electrostatic potential energy profile along the z direction within the perovskite layer at r = 0. (d) Electrostatic potential energy profile along the lateral direction on the top surface (z = 0) of the perovskite layer. The color palettes show the orientation of tip-sample bias voltage change from -2 V to 2 V. The red and blue curves represent the electrostatic response with and without surface states at the bottom surface of perovskite film, respectively.

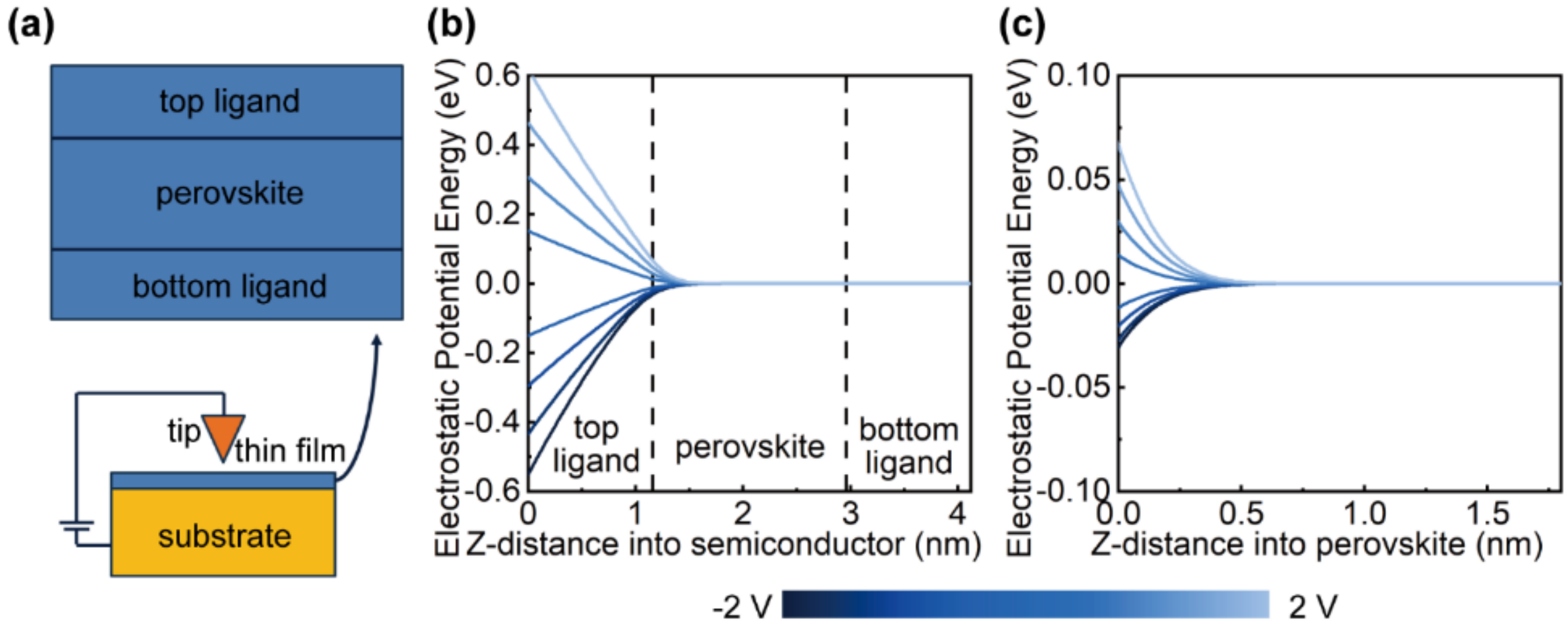

Figure S16. (a) Schematic plot of ligand-inclusive effective model for SEMITIP simulation. Both ligand layers were assigned the same dielectric constant as the perovskite layer, and their thicknesses were treated as effective electrostatic thickness based on a series-capacitor approximation. (b) Electrostatic potential energy profile along z-direction across the ligand/perovskite/ligand stack film. (c) Enlarged view of the electrostatic potential energy profile along z-direction within the perovskite layer only. The color palette shows the orientation of tip-sample bias voltage change from -2 V to 2 V.

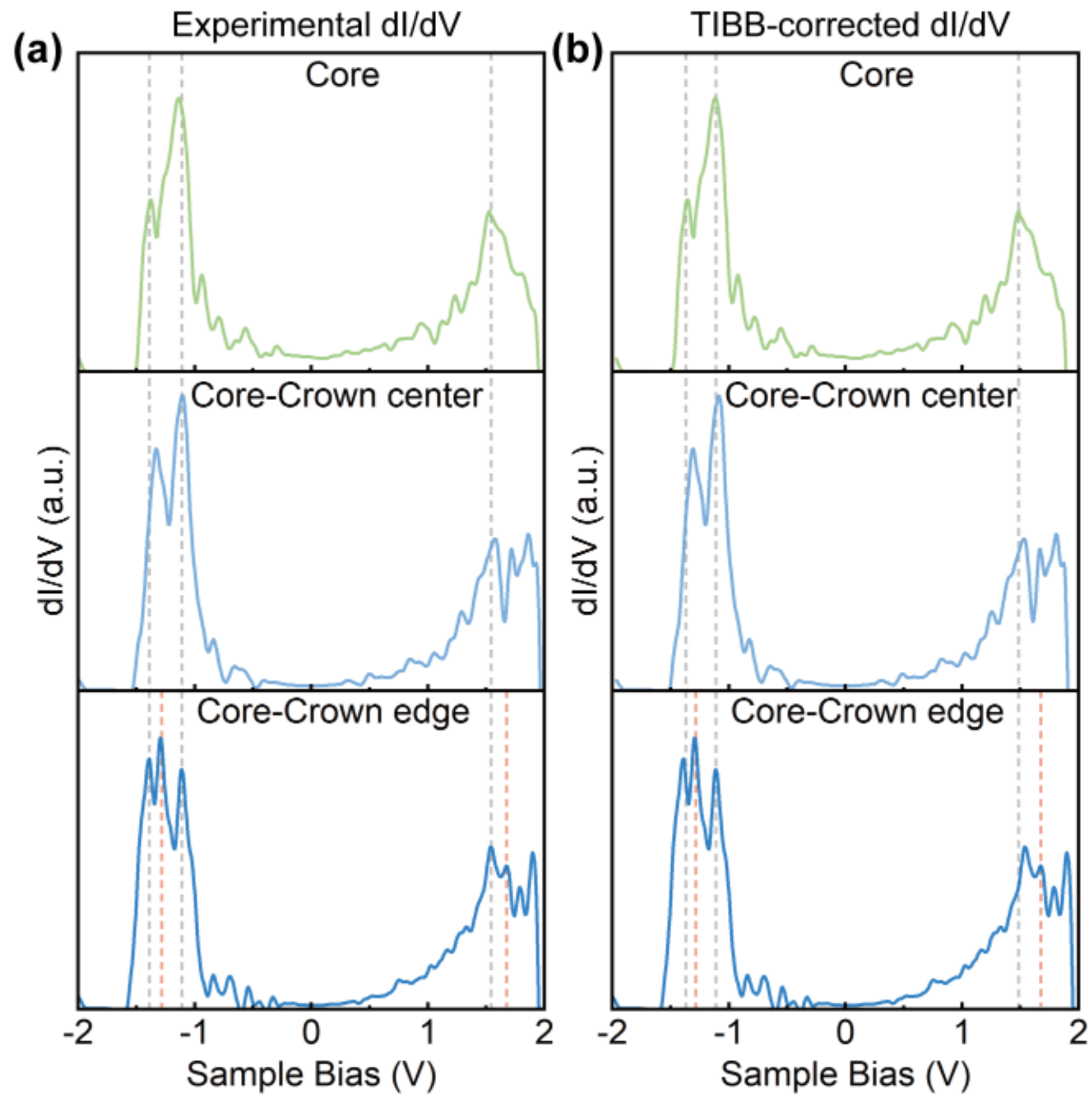

Figure S17. (a) dI/dV spectra acquired from center of core NPLs and both center and edge of core-crown NPLs. (b) Corresponding dI/dV spectra acquired from center of core NPLs and both center and edge of core-crown NPLs after TIBB correction based on the electrostatic potential energy values calculated from ligand-inclusive effective model.

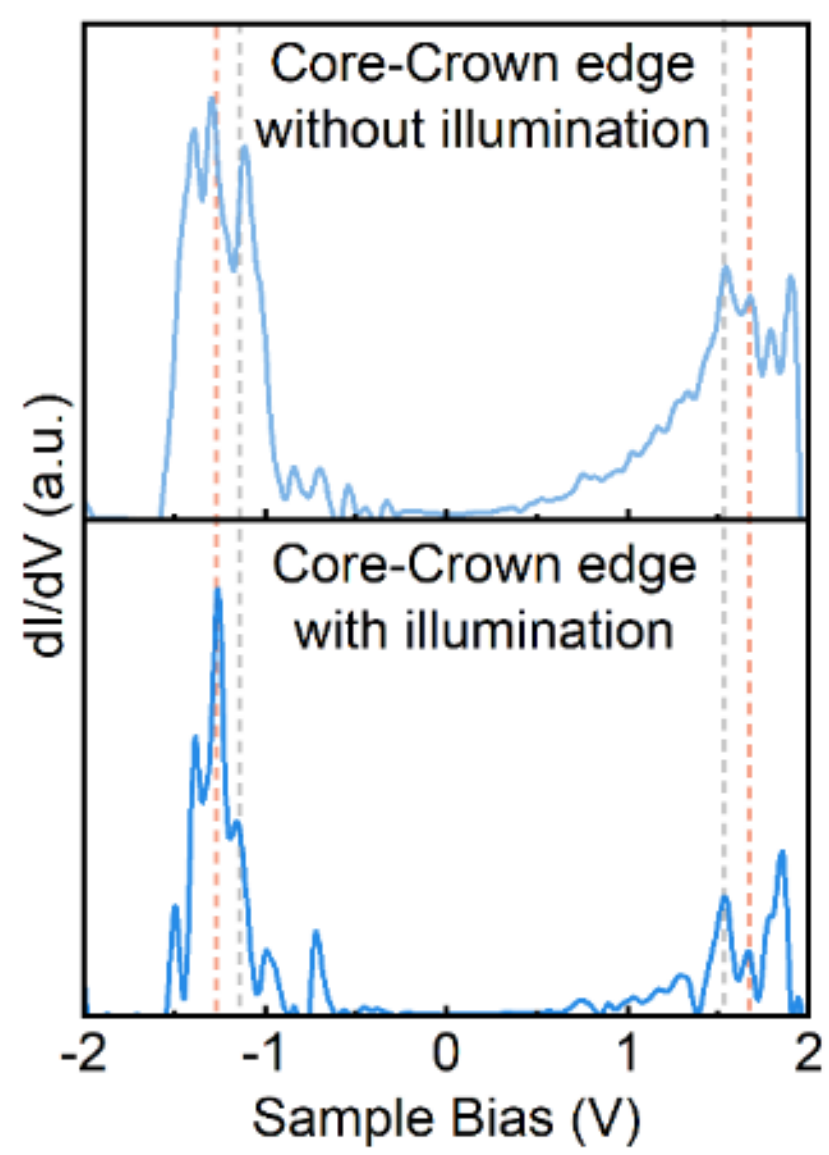

Figure S18. dI/dV spectra acquired from the edge of core-crown NPLs with and without UV light illumination. The gray and orange dashed lines represent the VBM and CBM of $CsPbBr_3$ and $FAPbBr_3$, respectively.

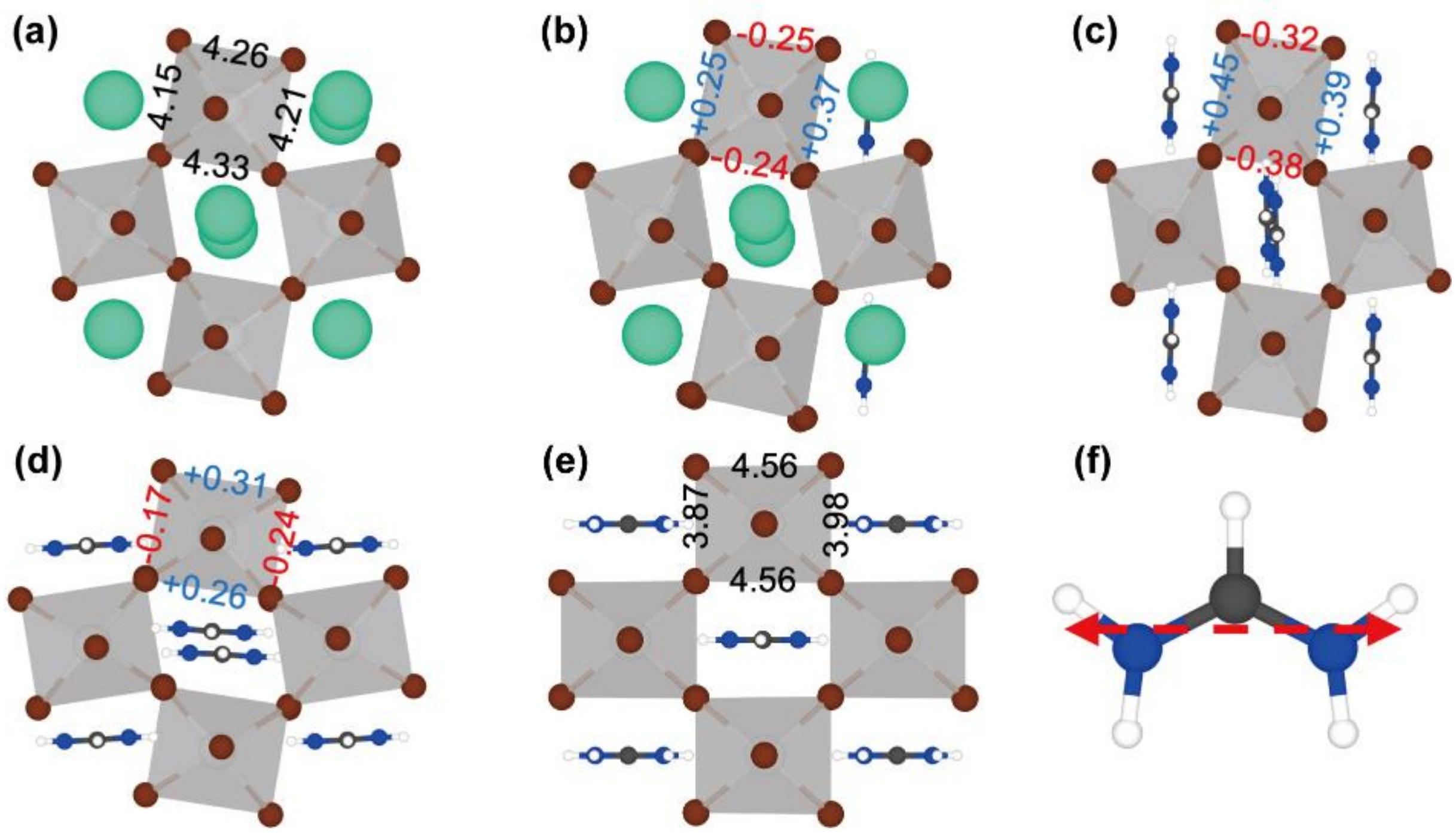

Figure S19. Visualization of all systems considered in theoretical computations, (a) orthorhombic $CsPbBr_3$ (0% FA), (b) 25% FA, (c) 100% FA (b-aligned), (d) 100% FA (a-aligned), and (e)

orthorhombic $FAPbBr_3$, and (f) the axis of alignment for the FA molecule. Measurements for polyhedral size of (a) and (e) are shown in Å. b-d) shows change in polyhedral size w.r.t. (a) in Å. Only one polyhedral is shown for clarity. Made with VESTA (13).

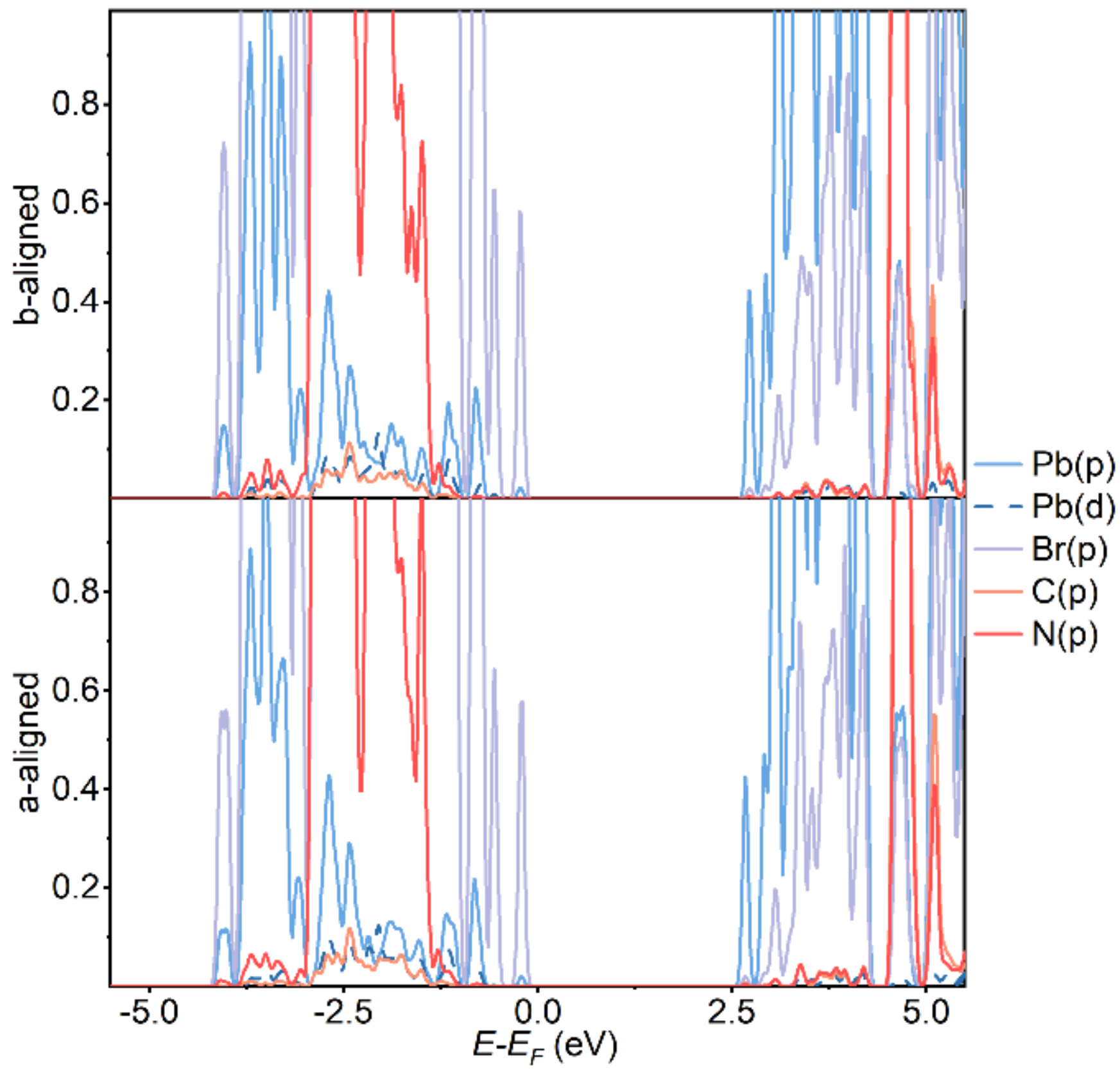

Figure S20. PDOS of a-aligned and b-aligned 100% FA systems.

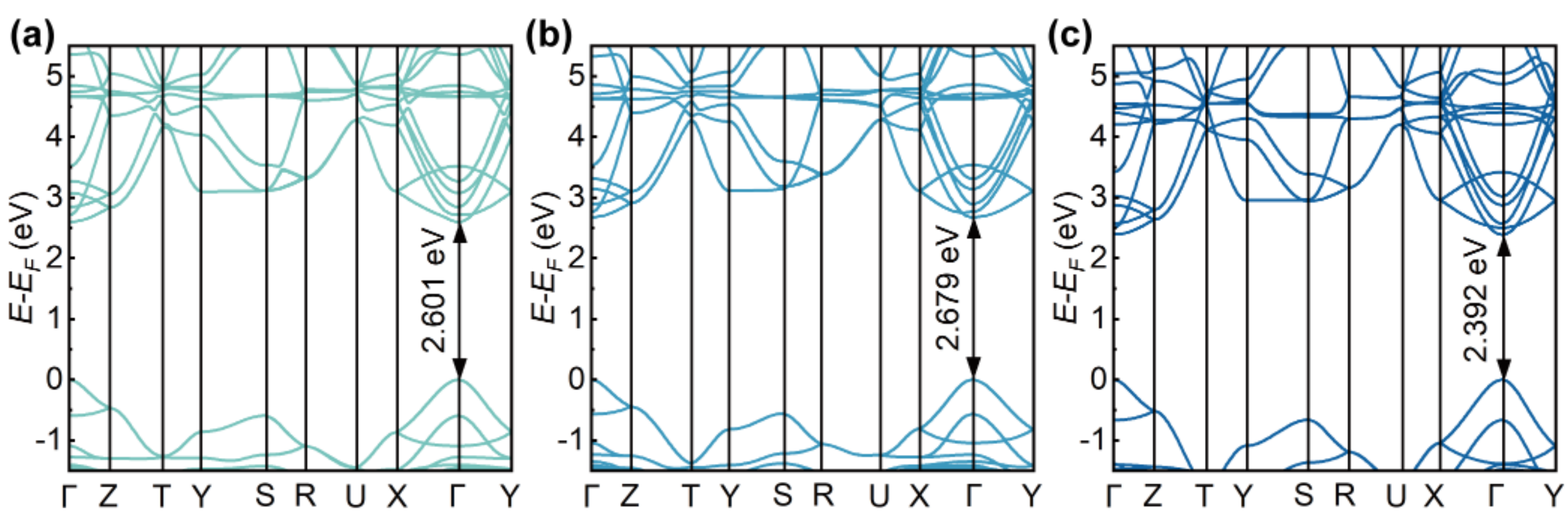

Figure S21. Band structure of (a) 100% FA (a-aligned) (b) 100% FA (b-aligned) (c) orthorhombic $FAPbBr_3$.

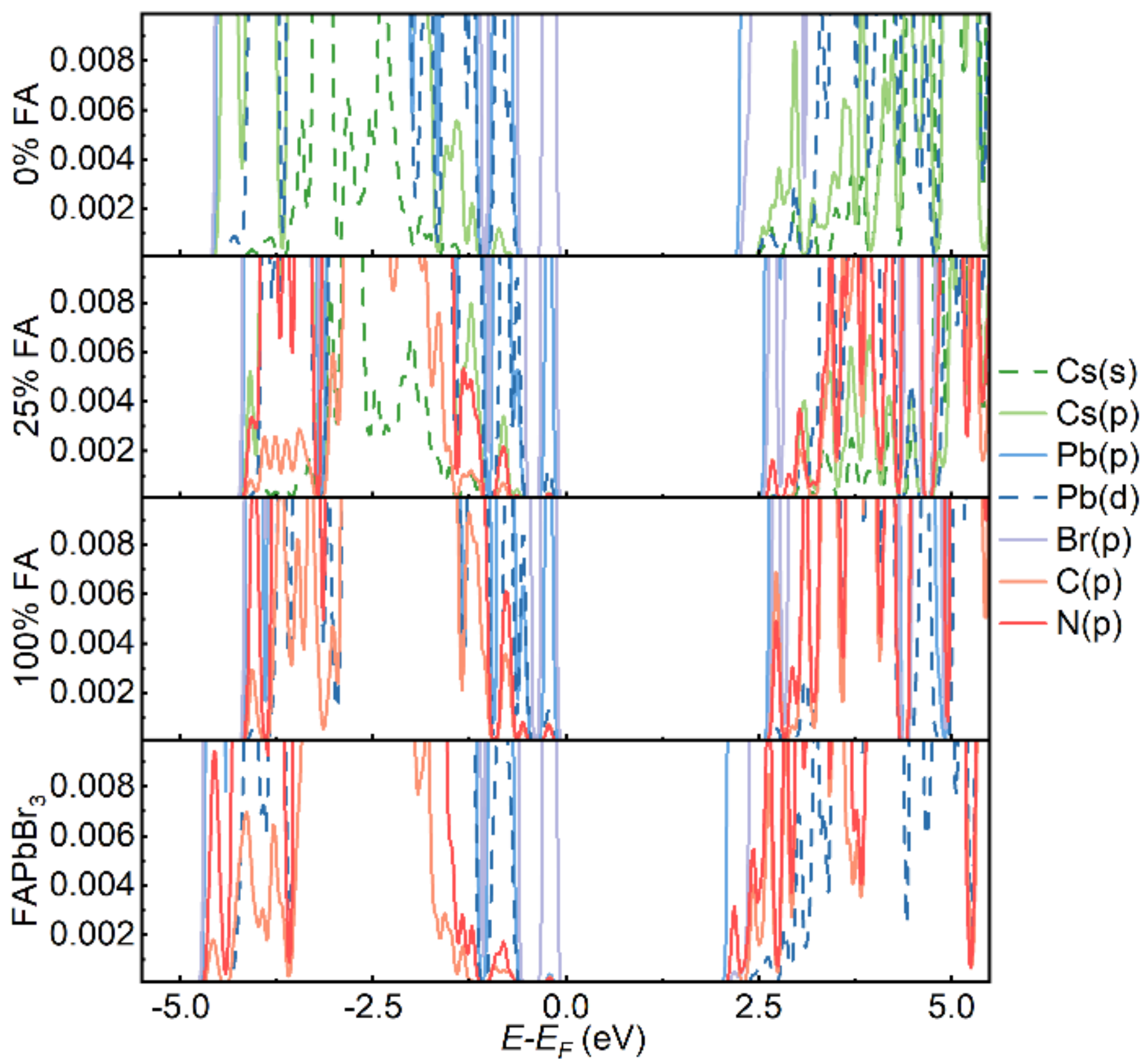

Figure S22. Zoomed version of Figure 4c highlighting contributions of different orbitals to VBM and CBM.

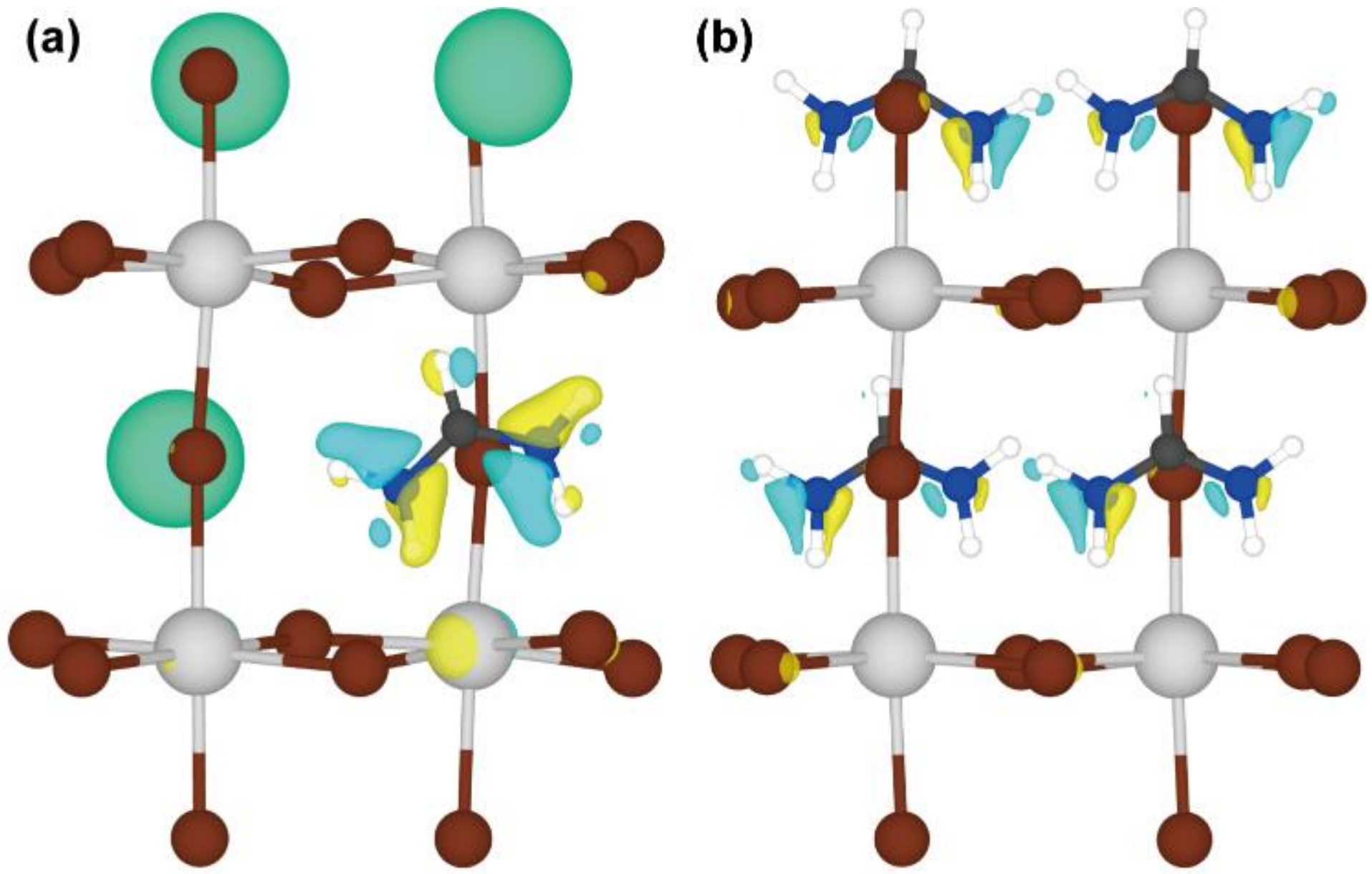

Figure S23. Charge density difference of the first triplet excited state and the ground state for (a) 25% FA and (b) 100% FA. Cyan represents negative charge accumulation and yellow represents positive charge accumulation. The isosurface cutoff is 0.08. Made with VESTA (13).

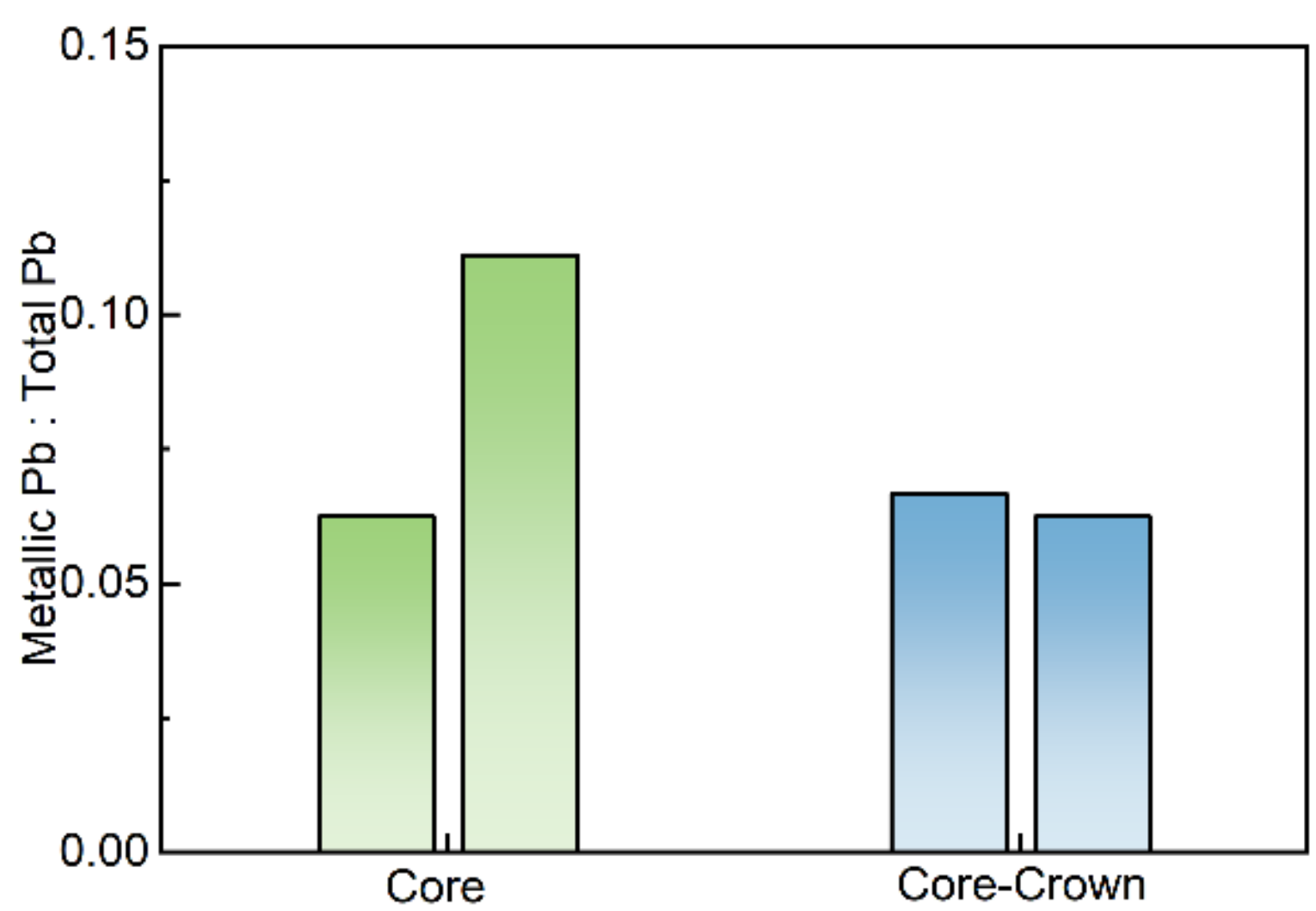

Figure S24. Metallic Pb : Total Pb atomic concentration ratio comparison of core and core-crown NPLs from XPS.

9. References

(1) Hamers, R. J. Atomic-Resolution Surface Spectroscopy with the Scanning Tunneling Microscope. *Annu. Rev. Phys. Chem.* **1989**, *40*, 531– 559.

(2) Feenstra, R. M. Semitip Software, **2011**; http://www.andrew.cmu.edu/user/feenstra/semitip_v6/

(3) Whitcher, T. J.; Gomes, L. C.; Zhao, D.; Bosman, M.; Chi, X.; Wang, Y.; Carvalho, A.; Hui, H. K.; Chang, Q.; Breese, M. B. H.Dual Phases of Crystalline and Electronic Structures in the Nanocrystalline Perovskite CsPbBr3. *NPG Asia Mater.* **2019**, *11*, 70.

(4) Kshirsagar, A. S.; Gangishetty, M. K. In situ controlled growth of strongly quantum confined $CsPbBr_3$/$FAPbBr_3$ core/crown nanoplatelets for blue light emitting diodes. *Adv. Opt. Mater*. **2023**, *11* (21), 2301343.

(5) Bohn, B. J.; Tong, Y.; Gramlich, M.; Lai, M. L.; Döblinger, M.; Wang, K.; Hoye, R. L. Z.; Müller-Buschbaum, P.; Stranks, S. D.; Urban, A. S.; Polavarapu, L.; Feldmann, J. Boosting Tunable Blue Luminescence of Halide Perovskite Nanoplatelets through Postsynthetic Surface Trap Repair. *Nano Lett.* **2018**, *18* (8), 5231– 5238.

(6) Peng, S.; Wen, Z.; Ye, T.; Xiao, X.; Wang, K.; Xia, J.; Sun, J.; Zhang, T.; Mei, G.; Liu, H.; Xu, B.; Li, X.; Chen, R.; Xing, G.; Wang, K.; Tang, Z. Effective Surface Ligand-Concentration Tuning of Deep-Blue Luminescent $FAPbBr_3$ Nanoplatelets with Enhanced Stability and Charge Transport. *ACS Appl. Mater. Interfaces* **2020**, *12*, 31863– 31874.

(7) Shih, M. C.; Li, S. S.; Hsieh, C. H.; Wang, Y. C.; Yang, H. D.; Chiu, Y. P.; Chang, C. S.; Chen, C. W. Spatially Resolved Imaging on Photocarrier Generations and Band Alignments at Perovskite/PbI2 Heterointerfaces of Perovskite Solar Cells by Light-Modulated Scanning Tunneling Microscopy. *Nano Lett.* **2017**, *17* (2), 1154– 1160.

(8) Huang, P. C.; Huang, S. K.; Lai, T. C.; Shih, M. C.; Hsu, H. C.; Chen, C. H.; Lin, C. C.; Chiang, C. H.; Lin, C. Y.; Tsukagoshi, K.; Chen, C. W.; Chiu, Y. P.; Tsay, S. F.; Wang, Y. C. Visualizing band alignment across 2D/3D perovskite heterointerfaces of solar cells with light-modulated scanning tunneling microscopy. *Nano Energy* **2021**, *89*, 106362.

(9) Wieghold, S.; Cope, E. M.; M?ller, G.; Shirato, N.; Guzelturk, B.; Rose, V.; Nienhaus, L. Stressing Halide Perovskites with Light and Electric Fields. *ACS Energy Lett.* **2022**, *7* (7), 2211– 2218.

(10) Yu, D.; Cao, F.; Qiu, X.; Chen, Y.; Zhang, Z.; Wang, G.; Mao, Y.; Zhong, J.; Su, C.; Huang, W.; Lee, T. W.; Xing, G. Violet/ultraviolet light-induced depassivation in halide perovskite solar cells. Nat. Commun. **2025**, 16, 11409.

(11) Kresse, G.; Hafner, J. Ab Initio Molecular Dynamics for Liquid Metals. *Phys. Rev. B: Condens. Matter Mater. Phys.* **1993**, *47*, 558– 561.

(12) Kresse, G.; Hafner, J. Ab initio molecular-dynamics simulation of the liquid-metalamorphous- semiconductor transition in germanium. *Phys. Rev. B: Condens. Matter Mater. Phys.* **1994**, *49*, 14251– 14269.

(13) Kresse, G.; Furthmüller, J. Efficiency of ab-initio total energy calculations for metals and semiconductors using a plane-wave basis set. *Comput. Mater. Sci.* **1996**, *6*, 15– 50.

(14) Kresse, G.; Furthmüller, J. Efficient iterative schemes for ab initio total-energy calculations using a plane-wave basis set. *Phys. Rev. B: Condens. Matter Mater. Phys.* **1996**, *54*, 11169– 11186.

(15) Kresse, G.; Joubert, D. From ultrasoft pseudopotentials to the projector augmented-wave method. *Phys. Rev. B: Condens. Matter Mater. Phys.* **1999**, *59*, 1758– 1775.

(16) Krukau, A. V.; Vydrov, O. A.; Izmaylov, A. F.; Scuseria, G. E. Influence of the exchange screening parameter on the performance of screened hybrid functionals. *J. Chem. Phys.* **2006**, *125*, 224106.

(17) Adamo, C.; Barone, V. Toward Reliable Density Functional Methods without Adjustable Parameters: The PBE0Model. *J. Chem. Phys.* **1999**, *110*, 6158-6170.

(18) Grimme, S.; Hansen, A.; Brandenburg, J. G.; Bannwarth, C. Dispersion-Corrected Mean-Field Electronic Structure Methods. *Chem. Rev.* **2016**, *116*, 5105– 5154.

(19) Monkhorst, H. J.; Pack, J. D. Special points for Brillouin-zone integrations. *Phys. Rev. B* **1976**, *13* (12), 5188– 5192.

(20) Ljungberg, M. P.; Mortensen, J. J.; Pettersson, L. G. M. An implementation of core level spectroscopies in a real space projector augmented wave density functional theory code. *J. Electron Spectrosc.* **2011**, *184*, 427.

(21) Wang, V.; Xu, N.; Liu, J. C.; Tang, G.; Geng, W. T. VASPKIT: A user-friendly interface facilitating high-throughput computing and analysis using VASP code. *Comput. Phys. Commun.* **2021**, *267*, 108033.

(22) Ganose, A. M.; Jackson, A. J.; Scanlon, D. O. Sumo: Command-Line Tools for Plotting and Analysis of Periodic Ab Initio Calculations. *J. Open Source Software* **2018**, *3* (28), 717.

(23) Momma, K.; Izumi, F. VESTA 3 for three-dimensional visualization of crystal, volumetric and morphology data. *J. Appl. Crystallogr.* **2011**, *44*, 1272– 1276.
